# How meristems shape plant architecture in cereals—Cereal Stem Cell Systems (CSCS) Consortium

**DOI:** 10.1093/plcell/koaf150

**Published:** 2025-06-20

**Authors:** Thomas Dresselhaus, Martina Balboni, Lea Berg, Anika Dolata, Frank Hochholdinger, Yongyu Huang, Guojing Jiang, Maria von Korff, Jia-Chi Ku, Karina van der Linde, Jan Maika, Cecilia Lara Mondragon, Michael T Raissig, Arp Schnittger, Thorsten Schnurbusch, Rüdiger Simon, Yvonne Stahl, Marja Timmermans, Venkatasubbu Thirulogachandar, Shuangshuang Zhao, Yaping Zhou

**Affiliations:** Institute of Plant Sciences, Cell Biology and Plant Biochemistry, University of Regensburg, Regensburg 93040, Germany; Institute of Plant Science and Microbiology, Developmental Biology, University of Hamburg, Hamburg 22609, Germany; Institute of Plant Sciences, University of Bern, Bern 3012, Switzerland; Institute of Molecular Biosciences, Goethe-University Frankfurt, Frankfurt am Main 60438, Germany; Crop Functional Genomics, University of Bonn, Bonn 53113, Germany; Leibnitz Institute of Plant Genetics and Crop Plant Research (IPK), Gatersleben 06466, Germany; Institute of Plant Sciences, Cell Biology and Plant Biochemistry, University of Regensburg, Regensburg 93040, Germany; Institute for Plant Genetics, Heinrich Heine University Düsseldorf, Düsseldorf 40225, Germany; Institute of Plant Sciences, Cell Biology and Plant Biochemistry, University of Regensburg, Regensburg 93040, Germany; Institute of Plant Sciences, Cell Biology and Plant Biochemistry, University of Regensburg, Regensburg 93040, Germany; Institute for Developmental Genetics, Heinrich Heine University Düsseldorf, Düsseldorf 40225, Germany; Center for Plant Molecular Biology, Eberhard Karls University Tübingen, Tübingen 72076, Germany; Institute of Plant Sciences, University of Bern, Bern 3012, Switzerland; Institute of Plant Science and Microbiology, Developmental Biology, University of Hamburg, Hamburg 22609, Germany; Leibnitz Institute of Plant Genetics and Crop Plant Research (IPK), Gatersleben 06466, Germany; Institute of Agricultural and Nutritional Sciences, Martin Luther University Halle-Wittenberg, Halle 06120, Germany; Institute for Developmental Genetics, Heinrich Heine University Düsseldorf, Düsseldorf 40225, Germany; Institute of Molecular Biosciences, Goethe-University Frankfurt, Frankfurt am Main 60438, Germany; Center for Plant Molecular Biology, Eberhard Karls University Tübingen, Tübingen 72076, Germany; Institute for Plant Genetics, Heinrich Heine University Düsseldorf, Düsseldorf 40225, Germany; Leibnitz Institute of Plant Genetics and Crop Plant Research (IPK), Gatersleben 06466, Germany; Crop Functional Genomics, University of Bonn, Bonn 53113, Germany

## Abstract

Meristems are major determinants of plant architecture, diversification, and acclimation to environmental stresses. Moreover, meristems play also a major role during crop domestication and are fundamentally important for the productivity of crop plants as they directly determine biomass and grain yield. While vegetative meristems shape the basic plant body plan and produce all above- and below-ground parts of plants, some vegetative meristems transit to reproductive meristems, forming sexual organs and germ cells. Most knowledge about plant meristems was generated using the model plant Arabidopsis. Compared with Arabidopsis, architecture of grass or cereals, including crops like maize, wheat, barley, rice and sorghum, is more complex: cereals produce additional organs like a coleoptile, seminal roots originating from the scutellar nodes in the embryo and shoot-borne crown roots as well as highly complex inflorescence meristems with meristem types absent in eudicots. Moreover, studies in cereals indicated that paradigms based on studies using Arabidopsis are not universally applicable. This review therefore aims to provide a comprehensive overview about the initiation, establishment, maintenance, and function of the various cereal meristems and their stem cell niches that shape our most important crop plants. Stem cell–like systems involved in leaf pattering and germline formation are also considered. The focus is also on the significant progress that has been made recently using novel tools to elucidate gene regulatory networks underlying the development and function of the various cereal meristems.

## Introduction

By Thomas Dresselhaus

Meristems produce all organs and shape the diversity of plant architecture. Moreover, they are key targets for cereal improvement (Lindsay et al. 2024). Meristems contain self-renewing pools of pluripotent stem cells that generate all plant organs, including leaves, stems, roots, and flowers. Within meristems, stem cells are embedded in stem cell niches (SCNs), which usually comprise slowly dividing organizing cells at the center that are surrounded by various types of stems cells (Hong and Fletcher 2023; Eljebbawi et al. 2024). Stem cells can undergo formative divisions and generate 2 types of daughter cells, with one cell maintaining stem cell identity while the other daughter cell starts to divide rapidly or differentiate, depending on its position. Maintaining plant meristems active over months or even during hundreds of years in woody plant species requires highly precise gene regulatory networks (GRNs). Such networks control among other features cell proliferation, self-renewal, and differentiation of daughter cells (Umeda et al. 2021). In addition to temporal, spatial developmental, and environmental cues, the activity of meristems and their stem cells in a plant´s body is integrated via long range signaling from distant organs, including the root, as well as highly variable environmental inputs. Environmental signals and age in particular appear to significantly modulate stem cell functions and the plant life cycle (Pfeiffer et al. 2017; Zeng et al. 2024).

During early embryogenesis, meristems and stem cell populations are specified from pluripotent cells to form and organize primary meristems. Meristems that are established post-embryonically, like axillary and intercalary meristems, inflorescence and lateral root meristems, are designated as secondary meristems. During embryogenesis in the model plant *Arabidopsis thaliana* (Arabidopsis), only the 2 main apical stem cell systems of the shoot and root (SAM and RAM) are specified that are required to determine the basic body plan and to produce all above- and below-ground parts of the plant. Cereal grasses contain larger, more complex, and additional meristems (Yu et al. 2016; McKim 2019; Wang et al. 2021a). Cereal embryos, for example, additionally develop seminal root meristems, whose number and outgrowth are also considered as an adaptive trait selected during domestication (Hochholdinger et al. 2018; Viana et al. 2022). In general, meristem identity in cereals is determined by the type of lateral organs they produce: for example, the vegetative SAM and RAM produce leaves and roots, respectively, while inflorescence meristems (IMs) are established after transition to a reproductive program to generate lateral indeterminate branch (BMs), spikelet (SM), and floret meristems (FMs), respectively, which ultimately generate flower organs. A rapid increase in meristem size is a first sign of SAM to IM transition (Bommert and Whipple 2018; Koppolu et al. 2022). Further cells with meristematic characters include stem cell-like cells named archespores (ARs) that are formed as precursors of germ cells, and their fate appears to be at least partially regulated by the same molecular players involved in SAM regulation (Zhao et al. 2017c). Similarly, meristematic cells lacking stem cell character (meristemoids) are generated in developing leaves as precursors of guard cells (Nunes et al. 2020).

Research in Arabidopsis has shown that meristem establishment, maintenance, and function are controlled by multiple pathways. SAM size and maintenance, for example, is regulated by an evolutionarily conserved negative feedback loop consisting of the mobile stem cell derived factor CLAVATA3 (CLV3), its receptor CLV1, and the homeodomain transcription factor (TF) WUSCHEL (WUS) (Demesa-Arevalo et al. 2024). WUS promotes stem cell fate and CLV3 expression but is suppressed by CLV signaling, which is further modulated by many additional actors. A similar conserved pathway exists in the RAM and also further *WUS-related homeobox* (*WOX*) genes that act in diverse stem cell systems (Sarkar et al. 2007; Song et al. 2021). For some of these key genes, homologs with similar functions appear to exist also in cereals. However, the expression pattern and function of many of these meristem genes is different in the grasses, indicating that stem cell signaling systems are diversified between all plant species. Functions of most homologs have not been studied in detail, and additional genes seem to play key roles in meristem development in cereals (Du et al. 2022; Koppolu et al. 2022). Gene redundancy and neo-functionalization further contributed to the tremendous difference of meristem function(s) between the eudicot model Arabidopsis, cereals, and other plant families. The goal of this review therefore is to provide a comprehensive overview about our current knowledge of the different types of cereal meristems and meristem-like cells, the underlying GRNs that regulate meristems, and their SCNs, highlighting also the potential of most recent technology that significantly advanced our understanding how cereal meristems shape grass architecture.

## Establishment of embryonic meristems and their SCNs

By Guojing Jiang and Thomas Dresselhaus

A crucial step during embryogenesis is the establishment of embryonic SCNs, the SAM and RAM, which are essential for postembryonic growth and organogenesis (Radoeva et al. 2019; Wu et al. 2024). While the molecular mechanisms underlying meristem formation have been extensively studied in the model plant Arabidopsis, the developmental processes in cereals, which differ significantly from eudicots, remain less understood. This chapter therefore explores the key aspects of meristem initiation in cereals, with a focus on the role of *WOX* genes and hormone signaling and the cereal-specific factors influencing SCN establishment.

### Cereals develop complex embryonic organs

**Figure 1.**
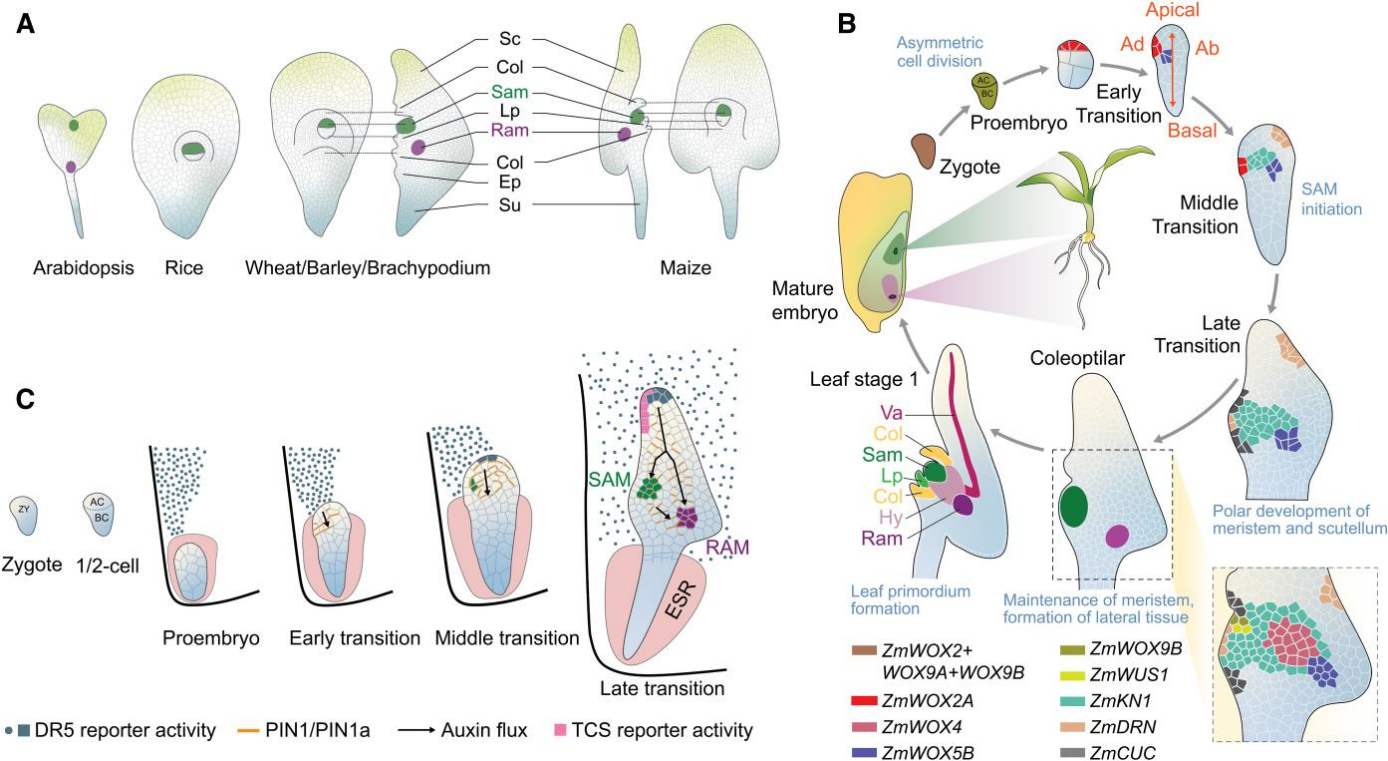
Meristem initiation and organ formation during cereal embryogenesis. Note that stages are not drawn to scale. **A)** Embryonic structures of Arabidopsis compared with different cereals. Shoot and root apical meristems are indicated. Partly front and side views are shown. **B)** Gene expression pattern regulating cell fate determination during early embryogenesis in cereals with maize as an example. Embryonic stages are indicated. Different colors mark embryonic cells expressing indicated genes. **C)** Auxin and CK responses during maize embryogenesis. Auxin flux is represented by black arrows. The DR5 and TCS reporter activities are marked in saxe-blue and pink, respectively. Abbreviations: Col, coleoptilar; Ep, epiblast; Hy, hypocotyl; Lp, leaf primordium; Ram, root apical meristem; Sam, shoot apical meristem; Sc, scutellum; Su, suspensor; Va, vasculature. C) Adapted from Chen et al. (2014).

Embryogenesis in flowering plants begins with the asymmetric division of the zygote establishing the apical-basal axis of the embryo (Armenta-Medina et al. 2021; Dresselhaus and Jürgens 2021). In Arabidopsis, the embryo structure is relatively simple, consisting of the typical embryonic tissues: primary shoot and root meristems, organized in a radial pattern with concentric layers of epidermal, ground, and vascular tissues, as well as 2 cotyledons (Fig. 1). The predictable and nearly invariant pattern of cell division in Arabidopsis embryos, along with their small size, makes them an ideal model for studying embryonic meristems. In contrast, grass embryos develop in a more complex and less ordered manner, exhibiting irregular cell division patterns and additional tissue layers. A major difference between grass and eudicot embryogenesis lies in the timing of meristem formation. In cereals such as rice, wheat, and maize, SAM formation occurs later, around 4 to 7 days post-fertilization, compared with approximately 3 days in Arabidopsis (Armenta-Medina et al. 2021). Moreover, cereal embryos develop additional organs, such as the scutellum, coleoptile, and epiblast, which are absent in eudicots (Itoh et al. 2005; Zhao et al. 2017b; Armenta-Medina et al. 2021). Embryos of different cereal species share similar patterning processes and tissue types during embryogenesis among the cereals: (1) the SAM initiates at the adaxial side and gives rise to multiple embryonic leaves; while (2) the scutellum, the single cotyledon of cereals, forms on the abaxial side, instead of the 2 cotyledons flanking the SAM as in eudicots; (3) the coleoptilar primordium extends from the adaxial region of the embryo to protect the developing SAM; and (4) the RAM originates at the base of the embryo proper, establishing the embryonic basal pole (Fig. 1, A and B) (Chandler et al. 2008; Radoeva et al. 2019; Armenta-Medina et al. 2021). The mechanisms of initiation and maintenance of these grass-specific embryonic organs have not been intensively studied. Some models describe scutellum and coleoptile as a fused but bipartite organ, where the scutellum represents the distal haustorial portion and the coleoptile serves as the proximal protective sheath (Kaplan and Specht 2022). Recent transcriptomic analyses in maize support this bipartite model, suggesting that both the scutellum and coleoptile are homologous to modified leaves (Wu et al. 2024).

### Transcriptional regulators of SCN formation

In contrast to Arabidopsis, cell division patterns during cereal embryogenesis are less predictable (Dresselhaus and Jürgens 2021). Following initial divisions, the embryo undergoes a critical transition from radial to bilateral symmetry, establishing its embryonic axis during the transition stage (Fig. 1B). At this stage, the embryo exhibits adaxial-abaxial polarity: the SAM forms at the adaxial side of embryo proper, while the scutellum develops at the abaxial side. This spatial arrangement differs from that in eudicot embryos, where 2 cotyledons are positioned symmetrically on either side of the SAM (Chandler et al. 2008; Radoeva et al. 2019). As development progresses into the coleoptilar stage, a coleoptilar ring begins to extend around the embryonic SAM, while the scutellum undergoes significant outgrowth. At this stage, both the embryonic SAM and RAM have fully differentiated, each containing well-defined SCNs. During leaf stage 1, the first leaf primordium is differentiated from the SAM, signifying that the major tissues required for the embryo's subsequent development have been established (Fig. 1B).

The molecular mechanisms governing the establishment of these embryonic meristems and SCNs in cereals remain largely unknown. Although both monocots and eudicots share conserved molecular players, the timing and expression pattern of these factors vary considerably. In Arabidopsis, *WOX2* and *WOX8* serve as early markers of apical and basal cell fates after zygote division (Haecker et al. 2004). However, in maize, *ZmWOX2A* is transiently expressed in the zygote and becomes significantly upregulated only at the late proembryo stage, coinciding with the initiation of meristem formation (Fig. 1B) (Nardmann et al. 2007). Likewise, the maize orthologs of *WOX8/9*, *ZmWOX9A/B/C*, exhibit limited expression until later stages of embryogenesis (Nardmann et al. 2007). Notably, *ZmWOX9B* is transiently expressed on the flanks of the embryonic SAM during the coleoptilar stage. Additionally, the expression of *ZmWUS1* and *ZmWOX5*, the maize orthologs of Arabidopsis *WUS* and *WOX5*, is delayed until the embryo contains more than 100 cells. These genes mark the shoot organizing center (OC) and root quiescent center (QC), respectively (Fig. 1B) (Zimmermann and Werr 2005; Zhao et al. 2017b). Moreover, *WOX* genes in cereals exhibit a more dynamic expression pattern during embryonic development, as exemplified by *OsWOX9* in rice (Tang et al. 2024): *OsWOX9A* and *OsWOX9C* play important roles in both the formation of the apical-basal axis and the initiation of stem cells. In summary, the relative delay in expression suggests that while the core mechanisms for niche establishment may be conserved, their temporal dynamics differ, potentially contributing to the distinct morphological traits of grass embryos.

In addition to the *WOX* genes, several other maize genes play critical roles in cell fate determination, meristem establishment, and maintenance during embryogenesis. These include *Dornröschen* (*DRN*), *Knotted1* (*KN1*), and *Cup-shaped cotyledon* (*CUC*), encoding TFs (Fig. 1B). The expression of *DRN* begins at the transition from the late proembryo to the early transition stage, and its function is closely associated with auxin signaling during early embryogenesis (Zimmermann and Werr 2007; Chandler et al. 2008). *KN1*, a key marker for the SAM during vegetative growth, starts to be expressed in early transition stage embryos. Its expression correlates with the differentiation of small, cytoplasmic-rich cells on the adaxial surface of the embryo body (Smith et al. 1995; Takacs et al. 2012). Importantly, *KN1* expression is not confined to the SAM but also extends to peripheral regions, indicating that *KN1* may be involved not only in meristem formation but also in the development of surrounding tissues. Similarly, *CUC* genes are expressed during the early transition stage and may contribute to establishing bilateral symmetry, working alongside *WUS1*, *WOX5*, and *DRN* homologs (Zimmermann and Werr 2005). Furthermore, *ALOG1*, a member of the Arabidopsis LSH1 and *Oryza* G1 (ALOG) TF family, is expressed at the boundaries between lateral organs and the developing SAM, suggesting its involvement in defining organ boundaries and contributing to the structural organization of the embryo (Takacs et al. 2012).

### Hormone signaling during embryonic meristem formation

Auxin and cytokinin (CK) signaling is integral to the establishment of embryonic SCNs in monocots. In maize, auxin signaling is delayed relative to Arabidopsis, where it initiates very early during embryogenesis. Auxin signaling in maize becomes evident during the transition stage, as shown by activity of the auxin signaling reporter *DR5* and auxin-dependent expression and intracellular localization of the auxin efflux carrier PINFORMED1 (ZmPIN1), which directs auxin flux from apical regions toward the future RAM (Fig. 1C) (Chen et al. 2014). This delayed auxin response appears crucial for establishing the apical-basal axis and coordinating the formation of SAM and RAM. Similarly, in the grass *Brachypodium distachyon* (Brachypodium), the role of auxin in embryogenesis follows a comparable delayed pattern (Hao et al. 2021). *DR5* auxin reporter activity is not detected in the early stages of Brachypodium embryogenesis; however, the key components of auxin transport, such as *BdPIN1a*, *BdPIN1b*, and *BdSoPIN1*, are expressed and polarized early on, suggesting that the system for auxin transport and response is in place, even if active signaling is delayed (O’Connor et al. 2014; Hao et al. 2021). Furthermore, the analysis of *PIN* genes in rice revealed that *OsPIN2*, *OsPIN5*, and *OsPIN8* are thus far the only known genes showing polarized expression patterns during embryogenesis (Itoh et al. 2016). This pattern contrasts with that observed in Arabidopsis embryos and other grasses, highlighting a species-specific divergence in the role of PIN-mediated auxin transport. Notably, unlike Arabidopsis, where auxin signaling is driven from within the embryo and the maternal integumental tissues connected to the embryonic suspensor (Robert et al. 2018), it is hypothesized that auxin signals required for embryonic patterning in maize originate from the adaxial endosperm, potentially influencing the asymmetric development of embryo structures (Forestan et al. 2010; Chen et al. 2014; Doll et al. 2017).

In addition to auxin, CK signaling appears to play a significant role in embryonic niche establishment in cereals. In maize, CK signaling, marked by activity of the transcriptional reporter *TCSv2*, begins at the late transition stage and is primarily localized to the adaxial surface of the protoderm and the SAM initiation site (Fig. 1C) (Chen et al. 2014). At the coleoptilar stage, CK signaling is detected in both shoot and root meristematic zones and, to a lesser extent, in the scutellum (Rijavec et al. 2011). These spatial and temporal patterns of CK activity complement auxin signaling in establishing the apical-basal axis and bilateral symmetry in maize embryos (Doll et al. 2017). In rice, 3 A-type CK response regulators (*OsRR5*, *OsRR6*, and *OsRR11*) are expressed in the apical-ventral region and function as negative regulators of CK signaling, thus contributing to SAM establishment before morphogenesis (Itoh et al. 2016). Their maize orthologue, *ABPH1*, is crucial for regulating SAM size and maintaining *PIN* expression, both of which are important for establishing phyllotaxy (Giulini et al. 2004; Lee et al. 2009). These regulators likely interact with auxin signaling to guide the proper formation of the SAM and ensure coordinated development during embryogenesis.

During early maize embryogenesis, neither *DR5* nor *TCSv2* signals were detected in the embryo-surrounding region (ESR) generated by the developing endosperm (Chen et al. 2014). ESR cells are small and contain dense cytoplasm, large nuclei, and abundant endoplasmic reticulum (Cosségal et al. 2007). The exact function of these cells remains unclear. Notably, the primary auxin response in maize kernels appears specific to the adaxial endosperm region apical to the ESR (Chen et al. 2014; Doll et al. 2017). It was hypothesized that the ESR may function as a buffer, shielding the early embryo from excessive auxin signals from the endosperm to regulate the timing of stem cell specification and lateral organ development not until the transition stage (Fig. 1C). As the maize embryo grows and progressively escapes the ESR and thus exposes its apical regions, hormonal responses—including auxin and CK signaling—become active coinciding with polar ZmPIN1 localization, likely mediating an apical-basal auxin gradient, which was suggested to be crucial for bilateral symmetry and meristem organization (Forestan et al. 2010; Chen et al. 2014). In terms of time, the formation of the SAM and RAM are directly linked to these processes (Doll et al. 2017). In conclusion, the dynamic interaction between the embryo and the ESR, along with the spatiotemporal regulation of auxin and CK signaling, appears fundamental for early embryonic patterning and proper establishment of meristems in maize and other cereals.

## Regulation of shoot apical meristem activity

By Marja Timmermans, Cecilia Lara Mondragon, Jan Maika, and Rüdiger Simon

**Figure 2.**
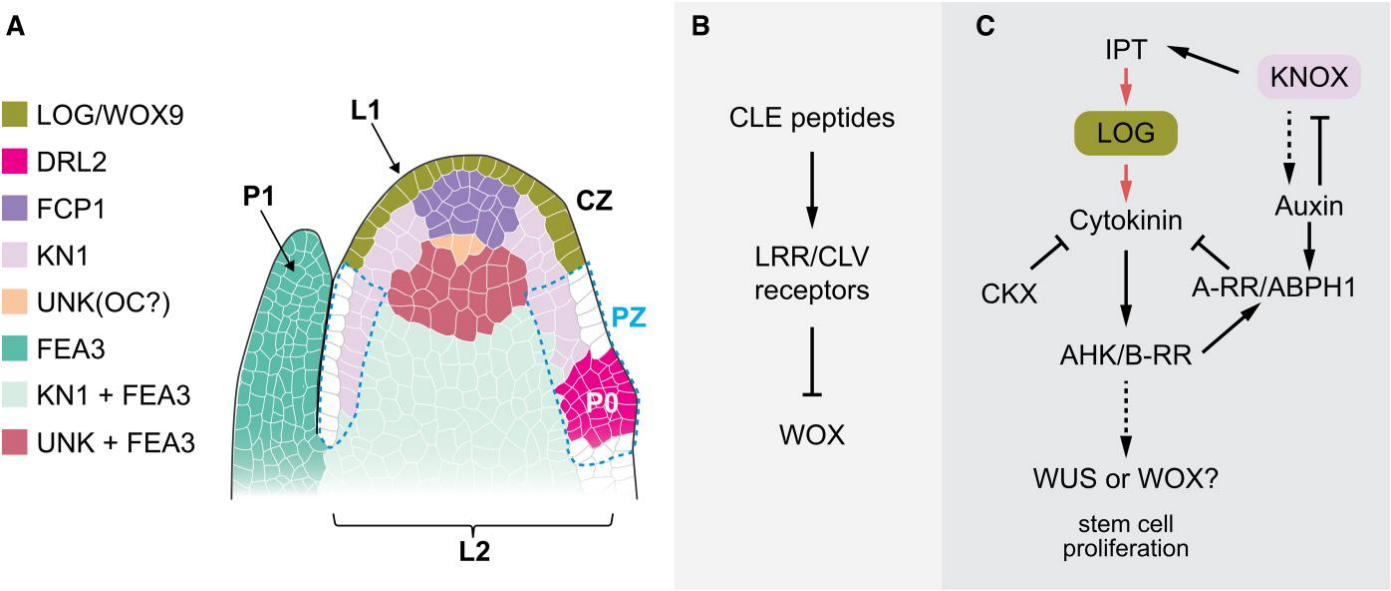
Organization of the vegetative SAM and the pathways controlling their activity in maize as a cereal model. **A)** The SAM is organized into discrete, functional domains, shown on the schematic on the left. Color-coded is a list of genes (see text for details) whose expression corresponds to functionally distinct domains in the SAM. Abbreviations: CZ, central zone; L1, epidermal layer; L2, inner cell layers; OC, putative organizing center; P0, incipient primordium; P1, primordium; PZ, peripheral zone. **B)** Simplified view of CLE-peptide mediated regulation of meristem activity. Multiple CLE-peptides are perceived by Leucine-Rich Repeat CLAVATA receptors and often negatively regulate *WOX* gene expression. **C)** Overview of the hormonal regulation of SAM homeostasis (see text for gene names). The schematic depicts known interactions between CK and auxin signaling pathways during SAM regulation. Red arrows represent metabolic pathways, while black arrows represent genetic interactions (full, validated interaction; dotted, putative interaction).

After its establishment during embryogenesis, the SAM, positioned at the growing shoot tip, is responsible for production of all aerial parts of a plant. Reflecting its 2 essential functions in post-embryonic growth—self-maintenance and organogenesis—the SAM is organized into at least 3 functional zones: the peripheral zone (PZ), where new organs form; the central zone (CZ), containing the reservoir of stems cells; and the organizing center (OC), which acts as a signaling center coordinating meristem activities (Fig. 2A). Superimposed on this zonation are discrete lineage layers (L) constituting the epidermis (L1) and depending on the species, 1 or more subepidermal tissues. SAM activity is tightly regulated by multiple, often interlinked molecular pathways that balance stem cell maintenance and organ production. These pathways reveal elements conserved across flowering plants, as well as adaptations unique to cereals. Below, we discuss mechanisms coordinating SAM maintenance, highlighting regulatory features that distinguish grasses.

### Diversification in the CLV-WUS signaling pathway

Stem cell maintenance in the SAM is well-studied in Arabidopsis. Here, the stem cell population in the CZ at the meristem tip is regulated by 2 key signaling centers: the L1 and the OC. The latter located directly below the stem cells is defined by expression of *WUS*. WUS protein moves via plasmodesmata to the cells at the tip of the CZ, where it promotes stem cell fate and induces expression of the secreted peptide CLV3. CLV3, in turn, is perceived by plasma membrane localized leucine-rich repeat (LRR) receptors, such as the LRR-kinase CLV1 and its co-receptor CIK, or the LRR-protein CLV2 in complex with the pseudokinase CORYNE (CRN). CLV signaling results in downregulation of *WUS*, creating a negative feedback loop that maintains stem cell number (Demesa-Arevalo et al. 2024). In addition, signals derived from the L1 anchor the OC to the SAM tip, ensuring proper spatial organization despite ongoing growth. The phytohormone CK influences the apical–basal positioning of the OC by promoting *WUS*, while the mobile small RNAs miR394 and miR171 render only cells at the tip of the CZ competent to respond to *WUS* and acquire stem cell fate (Chickarmane et al. 2012; Knauer et al. 2013; Yadav et al. 2013; Gruel et al. 2016; Han et al. 2020).

Core elements of the *CLV-WUS* signaling pathway (Fig. 2B) are conserved in cereal crops. However, along with the emergence of novel meristem types in the grasses, these key components have undergone extensive subfunctionalization and show significant changes in their spatial distribution. Perhaps most striking, the rice and maize orthologs of *WUS* do not display the canonical OC-specific pattern of expression in the vegetative SAM. While *WUS* expression in floral meristems mirrors that of the Arabidopsis counterpart (Je et al. 2016; Chen et al. 2021; Schlegel et al. 2021), expression in the vegetative SAM is absent from the meristem core and instead is associated with leaf emergence (Nardmann and Werr 2006; Knauer et al. 2019; Tanaka and Hirano 2020). This change in expression has 2 important implications: first, *WUS* signaling has potentially been co-opted to regulate different developmental programs independent of meristem maintenance; and second, alternate players must control meristem homeostasis during vegetative development.

The WUSCHEL-LIKE HOMEOBOX TF OsWOX4 may fulfill this function in rice (Ohmori et al. 2013). WOX4 orthologs are known to function in vascular development in other species (Ji et al. 2010; Etchells et al. 2013; Dai et al. 2023), but *Os*WOX4 shows expression throughout the rice apex, and its repression via RNAi leads to a decrease in vegetative meristem size (Ohmori et al. 2013). In maize, *Zm*WOX9 might serve this role (Knauer et al. 2019). Both *ZmWOX9B* and *ZmWOX9C* are expressed specifically in the epidermis of the CZ (Fig. 2A), presenting the intriguing possibility that (i) *Zm*WOX9 forms an inward-directed signal that both promotes stem cell identity and anchors the stem cell zone to the SAM tip, and (ii) the WUS/WOX-based signaling center is displaced in the maize vegetative SAM. Genetic confirmation for this idea is still pending, but the expression pattern of *ZmFCP1,* which encodes a CLV3/ENDOSPERM SURROUNDING REGION (CLE) peptide, is certainly intriguing in this regard. *ZmFCP1* is expressed directly below the *ZmWOX9* expression domain in the CZ in a pattern partially overlapping that of its putative receptors *ZmFEA2* and *ZmFEA3* (Je et al. 2016, 2018; Knauer et al. 2019). Although expression of a *WOX* gene has not been identified in the position of the canonical OC, a central signaling center that coordinates cell fates in the vegetative maize SAM may still exist. A gene of unknown function along with genes involved in hormone-, redox-, and sugar-based signaling identify a domain in the maize vegetative SAM equivalent in position and function to the Arabidopsis OC (Nardmann and Werr 2006; Knauer et al. 2019). Moreover, mutations in several of these genes affect meristem size, indicative of conservation also at the functional level (Knauer et al. 2019).

The *CLV* side of the *CLV-WUS* signaling pathway is similarly characterized by numerous elaborations and marked changes in spatiotemporal expression. For example, commensurate with the distinctive WUS localization, the maize *AtCLV1* ortholog *thick tassel dwarf1* (*TD1*) is expressed in leaf primordia but not the vegetative SAM (Bommert et al. 2005). Also, the rice ortholog *FLORAL ORGAN NUMBER 1* (*FON1*), while expressed in the SAM, shows a much broader domain of expression (Suzaki et al. 2004; Schlegel et al. 2021). Moreover, FON1 and its putative ligand FON2 control meristem size during vegetative, reproductive, and floral development. However, respective mutants display only subtle changes in vegetative SAM size, whereas FMs are greatly enlarged (Suzaki et al. 2004, 2006; Chu et al. 2006). Three additional CLE-peptides, *FON2-LIKE CLEPROTEIN1* (*FCP1*), *FCP2*, and *FON2 SPARE1* (*FOS1*), redundantly control SAM homeostasis independently from *FON1* during vegetative growth through regulation of *WOX4* (Suzaki et al. 2009; Ohmori et al. 2013).

Such elaborations seem to exist also in Triticeae crops, such as barley or wheat (Wang et al. 2023; Vardanega et al. 2025), as well as in maize. In maize IMs, where *ZmWUS1* defines the OC, its expression is tightly regulated by the combined actions of TD1, FEA3, the CLV2 orthologue FEA2, and CRN, which all seem to act as receptors for *Zm*FCP1 (Bommert et al. 2005, 2013; Je et al. 2016, 2018; Chen et al. 2021; Liu et al. 2021). In addition, *Zm*CLE7 activates a signaling cascade via FEA2 that incorporates the heterotrimeric G-protein α-subunit COMPACT TASSEL2 (CT2) and the β-subunit *Zm*GB1 (Bommert et al. 2013; Je et al. 2018; Wu et al. 2020). Mutations in any one of these signaling components minimally affect the vegetative SAM but lead to fasciation of IMs, particularly the ear, due to the overproliferation of stem cells linked to elevated expression of ZmWUS1. It is tempting to postulate that elaboration of the CLV pathway contributes to the selected robustness in cereals—for example, by more precisely tuning downstream molecular circuitries across meristem types while at the same time increasing signaling potential, allowing to accommodate a wider spectrum of intrinsic or environmental inputs.

### Hormonal control of SAM activity

Meristem activity further relies on a delicate balance between CK and auxin signaling. CK produced in the CZ maintains meristem identity by promoting stem cell proliferation and inhibiting their differentiation. The bioavailability of CKs at the shoot apex is closely regulated. LONELY GUY (LOG) enzymes, located in the epidermis (Fig. 2C), convert inactive CK nucleotides into their bioactive free-base form, possibly generating an apical-basal active CK gradient as in Arabidopsis (Chickarmane et al. 2012). Loss of *LOG* function in maize and rice results in meristem termination (Kurakawa et al. 2007; Knauer et al. 2019). Conversely, CK oxidase/dehydrogenases (CKX) irreversibly inactivate CKs, and reduced CKX activity, as conditioned by the rice *Gn1a* QTL, leads to an increase in meristem size due to increased CK levels (Ashikari et al. 2005).

CK signals through a histidine kinase (HK)-based, 2-component phosphorelay system and the activation of Type B response regulators (B-RRs). These TFs promote transcription of CK-responsive genes, including Type A-RRs. The latter, in turn, repress CK-responsive genes, creating a negative feedback loop that tempers and sensitizes the CK response (Ferreira and Kieber 2005). Loss of HK or Type-B RR function results in a decrease in meristem size (Worthen et al. 2019; Burr et al. 2020; Chun et al. 2023), whereas loss of function mutations in maize *ABPHYL1*, a Type A-RR, give rise to a large meristem phenotype and altered phyllotaxy (Giulini et al. 2004). In the maize inflorescence, CK signaling appears to directly control *ZmWUS1* transcription (Chen et al. 2024), suggesting it may serve as an L1-derived signal positioning the SCN also in this developmental context. Whether CK provides this function in the vegetative SAM, perhaps in conjunction with ZmWOX9, remains unclear. Notably, a novel CK receptor involving serine/threonine instead of histidine phosphorylation was recently identified in rice (Halawa et al. 2021). While the downstream targets are yet to be described, this finding points toward unexpected, potentially grass-specific diversity in CK signaling.

In contrast to CK, auxin promotes organogenesis. Auxin forms a feedforward system with its efflux carrier PIN1 that self-organizes into a periodic pattern of auxin/PIN1 maxima at the meristem PZ at which new organs form (Kuhlemeier 2007). Functional orthologs to PIN1 have been identified in several grass species, including maize, rice, barley, and Brachypodium (Xu et al. 2005; Gallavotti et al. 2008; O’Connor et al. 2017; Kirschner et al. 2018; Fusi et al. 2024). In addition, members of a distinct clade of PIN proteins, the so-called Sister-of-PIN1 (SoPIN1) clade, contribute to organ formation in grasses separately from PIN1 (O’Connor et al. 2017). Disruption of auxin biosynthesis, transport, or signaling affects overall plant architecture, including meristem activity, but particularly axillary meristem activity and thereby inflorescence architecture (for more details, see the section on inflorescences below). Additional evidence connecting auxin to meristem homeostasis comes from a genome-wide association study, which identified *ZmLAX2*, an auxin influx carrier, as associated with shoot meristem morphological variation. Similar to PIN1, *ZmLAX2* accumulates in the developing leaf primordia and changes in its expression are linked to meristem size (Leiboff et al. 2015). It will now be important to elucidate whether genes expressed in the earliest leaf primordium (P0; Fig. 2A) like *DROOPING LEAF1*/2 (*DRL1/2*) encoding maize CRABS CLAW co-orthologs of the YABBY family of transcriptional regulators (Strable et al. 2017) are indeed activated at certain auxin thresholds.

### Additional pathways regulating SAM homeostasis

Given their antagonistic roles, a spatial integration of auxin and CK signaling pathways is necessary to balance organ formation with meristem maintenance. The homeodomain TF KN1 exemplifies 1 of several mechanisms connecting the 2 signaling pathways (Schaller et al. 2015). KN1 promotes the expression of *ISOPENTENYL TRANSFERASE* (*IPT*) genes, which encode enzymes involved in CK biosynthesis (Ikeda et al. 2019). In turn, *KN1* expression is suppressed by auxin. Accordingly, *KN1* is broadly expressed in the SAM but excluded from the L1 as well as developing leaf primordia (Fig. 2A) (Kerstetter et al. 1997). Loss of KN1 function in maize underscores its importance in meristem homeostasis, with phenotypes ranging from a reduced meristem size to meristem termination, depending on the genetic background. However, beyond its role in CK regulation, KN1 overwhelmingly targets developmental regulators, including many auxin-related genes that function in developing organ primordia (Bolduc et al. 2012; Knauer et al. 2019; Satterlee et al. 2020). As such, KN1 is proposed to generate a state of default repression that safeguards cells in the meristem from erroneously activating the differentiation program. This regulatory role is further highlighted in the *hooded* mutant in barley, where overexpression of the KN1-like homeobox gene *BKn3* stimulates organ outgrowth, in part by modulation of auxin dynamics (Richardson et al. 2016).

## Function and maintenance of root apical meristems

By Anika Dolata and Yvonne Stahl

Plant roots provide anchorage in the soil and access to essential nutrients and water, which is crucial for the formation of all above-ground tissues. The root systems need to rapidly adapt to changing environmental conditions, requiring fine-tuned growth and development. This is ensured by the presence and maintenance of stem cells in plant root meristems, which are located at the tip of the RAM in a SCN.

### The root SCN

**Figure 3.**
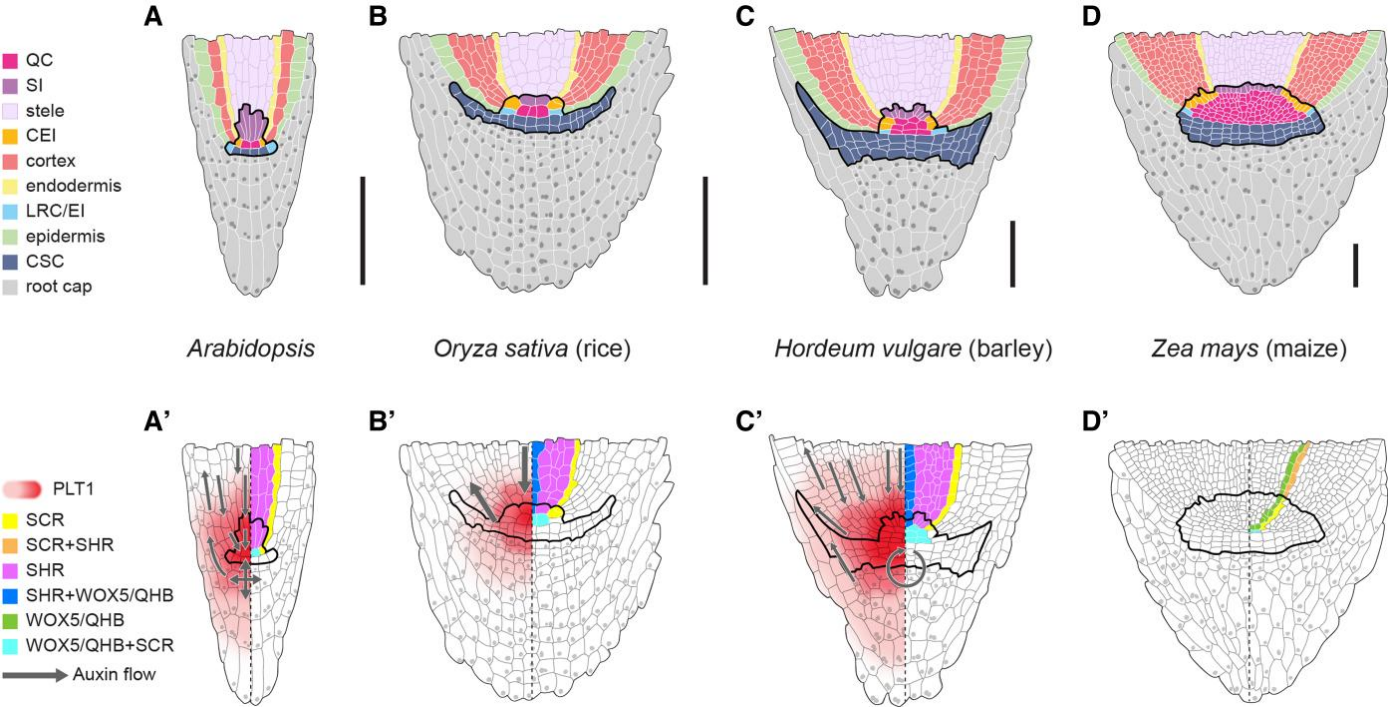
Root apical meristems (RAMs) in crops. **A to D)** Schematic overview of different cell types in the RAMs of Arabidopsis (A), rice (B), barley (C) and maize (D) as indicated. Abbreviations: CEI, cortex/endodermis initials; CSC, columella stem cells; LRC/EI, lateral root cap/epidermis initials; QC, quiescent center; SI, stele initials. **A′ to D′)** Expression patterns of PLETHORA 1 (PLT1), SCARECROW (SCR), SHORTROOT (SHR), WUSCHEL-RELATED HOMEOBOX 5/QHB (WOX5/QHB) and their overlapping pattern as indicated in the RAMs of Arabidopsis (A′), rice (B′), barley (C′) and maize (D′). Auxin flow is indicated with gray arrows.

The root SCN of Arabidopsis contains on average 4 to 8 pluripotent stem cells with low mitotic activity, called the quiescent center (QC) (Dolan et al. 1993; Baum et al. 2002; Lu et al. 2021). These cells divide asymmetrically and give rise to a surrounding group of stem cells, namely the stele initials, cortex/endodermis initials, epidermis/lateral root cap initials, and columella stem cells (Fig. 3A) (Dolan et al. 1993; van den Berg et al. 1997; Benfey and Scheres 2000). The daughter cells of these initials give rise to all the different tissues of the root. Even though they can be very different in size, the general architecture of the root meristem is conserved between monocots and eudicots. However, differences can be observed in cell size and number. Figure 3, B to D illustrates that the QC contains on average 3 to 4 cells in rice, around 30 cells in barley, and 800 to 1,200 cells in maize (Jiang et al. 2003; Kamiya et al. 2003b; Jiang and Feldman 2005; Kirschner et al. 2017). It has been hypothesized that in these bigger stem cell pools, both symmetric as well as asymmetric divisions, are necessary for maintaining stem cell homeostasis (García-Gómez et al. 2020).

### Hormonal control of root meristem activity

In the RAM of Arabidopsis, numerous players involved in SCN maintenance have been identified, including phytohormones such as auxin and CK, reactive oxygen species (ROS), as well as various key TFs (Drisch and Stahl 2015; Strotmann and Stahl 2021). Together with other hormones like CK, auxin has been shown to influence root meristem maintenance and root growth (Drisch and Stahl 2015). Auxin distribution within the plant is mediated by 2 distinct transport systems: a rapid, nondirectional stream in the phloem, and polar cell-to-cell transport. The latter relies on the activity of members of the *PIN* gene family (reviewed in Adamowski and Friml 2015). In Arabidopsis roots, PIN1–4 and PIN7 establish a so-called “reflux loop” of auxin, thereby exhibiting partial redundancy in their function (Blilou et al. 2005). In cereals, homologs of *PIN* family members have been described in various species, including barley (Kirschner et al. 2018), rice (Wang et al. 2009), and maize (Forestan et al. 2012). The zinc-finger homeobox gene *OsZHD2* from rice, which is primarily expressed in the SAM and root tips, enhances root meristem activity by inducing ethylene accumulation, ultimately leading to increased auxin biosynthesis (Yoon et al. 2020). A mutation in *OsZHD2* results in longer seminal and lateral roots caused by increased root meristem activity, and *OsZHD2* overexpressing plants displayed improved grain yield under low nutrient and paddy field conditions. *LATERAL ROOT PRIMORDIA1* (*LRP1*) encodes a transcriptional activator in maize that is regulated by the AUXIN/INDOLE-3-ACETIC ACID (Aux/IAA) protein ROOTLESS WITH UNDETECTABLE MERISTEM1 (RUM1; see also below). *LRP1* expression is auxin-inducible and is restricted to early root primordia and meristems, where its protein localizes to the nucleus and activates downstream gene expression. Repression of *LRP1* by RUM1, combined with its specific expression in root meristems, indicates a role in maize root development through the RUM1-dependent auxin signaling pathway (Zhang et al. 2015). Rice MERISTEM ACTIVITYLESS (MAL), containing a RING-H2 finger domain, regulates the viability of meristem cells following the initiation of root primordia, mediated by CK signaling (Jiang et al. 2020).

### Regulation of the root meristem appears largely conserved

Despite the wealth of knowledge about molecular regulation of the Arabidopsis root meristem, the molecular regulation in cereals still remains largely enigmatic (Fig. 3, A′ to D′). However, a homolog of the key QC regulator in Arabidopsis WOX5 has been characterized already in rice as QUIESCENT CENTER-SPECIFIC HOMEOBOX (QHB). *QHB* is primarily expressed in the rice QC, and QHB has been proposed to regulate the specification and maintenance of the QC in a mechanism that is similar to that for WUS in the SAM (Kamiya et al. 2003b). Furthermore, the PLETHORA1 (PLT1) TF, a member of the auxin-dependent AP2-type PLETHORA family, is a known important regulator of the Arabidopsis root SCN (Aida et al. 2004; Galinha et al. 2007). In rice, PLT1 nonredundantly promotes post-embryonic root development as well as crown-root development. Furthermore, it functions in regulation of rice root development by activation of auxin biosynthesis genes, such as *YUC1* and *YUC3* (Garg et al. 2022). Additionally, expression of the wheat PLT1 homolog, along with PLT3 and PLT5, is hypothesized to be linked to root meristem arrest (Gabay et al. 2023). In barley, a PLT1 homolog has been described (HvPLT1), exhibiting a similar expression pattern as in Arabidopsis (Kirschner et al. 2018) indicating a conserved function of these TFs in different plant species. In Arabidopsis, cortex and endodermis cell fates are established by the GRAS TFs SHORTROOT (SHR) and SCARECROW (SCR). SHR protein moves from the stele to the endodermis, where it interacts with SCR, thereby preventing further SHR movement. SCR/SHR interaction is required for establishing endodermis cell fate by regulating an asymmetric division of the endodermis/cortex initial, and for QC-specification and maintenance (Lim et al. 2000; Nakajima et al. 2001; Gallagher et al. 2004; Gallagher and Benfey 2009). SHR homologs from Brachypodium and rice can restore endodermal cell fate in Arabidopsis *shr* mutants, the maize SCR homolog can rescue defects in Arabidopsis *scr* mutant roots (Lim et al. 2005; Wu et al. 2014), and interaction studies between rice SHR and rice SCR further supported the hypothesis of a conserved function of these TFs (Cui et al. 2007). However, monocot SHRs can move from the stele to ground tissue when expressed in Arabidopsis without being limited to the endodermis by interaction with SCR from Arabidopsis. This movement then leads to the formation of additional ground tissue layers in Arabidopsis, indicating a potential role of SHR mobility in promoting the often high number of cortex cell layers in monocots (Wu et al. 2014). The maize *scr* mutant only partially resembles the Arabidopsis *scr* mutant phenotype pointing at potential redundancy of maize *SCR* genes (Slewinski et al. 2012). In barley, the expression patterns of *HvSHR* and *HvSCR* resemble those observed in Arabidopsis, once again indicating a conserved mechanism regulating cortex and endodermis specification through *SHR* and *SCR* (Kirschner et al. 2017). Another key regulator of rice root meristem activity and cell proliferation is the F-box protein *OsSHORT PRIMARY ROOT* (*OsSHPR*), which acts as an E3 ubiquitin ligase (Ma et al. 2023). SHPR forms a complex with the S-Phase Kinase-Associated Protein 1-like proteins SKP1-like1 and -20, and interacts with SEUSS-LIKE (*Os*SLK) in the nucleus to promote its degradation. Overexpression of *OsSLK* results in shorter primary roots, similar to SHPR loss-of-function mutants.

Alongside the above-mentioned TFs, the Arabidopsis CLE peptides CLV3 and CLE40 function in pathways essential for shoot and root meristem maintenance, respectively (Clark 1997; Fletcher et al. 1999; Brand et al. 2000; Schoof et al. 2000; Hobe et al. 2003). Corresponding homologs have been identified in rice, namely FLORAL ORGAN NUMBER2 (FON2) and FON2-LIKE CLE PROTEIN1 (FCP1) (Suzaki et al. 2006, 2008; Chu et al. 2013). In barley, *HvCLE402* encodes a CLE peptide closely related to FCP1 (The International Barley Genome Sequencing Consortium 2012; Kirschner et al. 2017). Incubation with *Hv*CLE402 peptides can restrict maintenance of the proximal root meristem in barley; however, the distal meristem (columella stem cells and the columella itself) is not strongly affected by this peptide signaling pathway, contrasting findings from Arabidopsis (Kirschner et al. 2017). In addition to CLE peptides, the 5 amino acid PEPTIDE1 (PEP1), encoded by *OsPEP1* in rice is involved in root development. *OsPEP1* is expressed in root tissues, especially in root cap and epidermis cells in the maturation zone. *Os*PEP1 inhibits primary root growth, making it an interesting candidate for regulating root development via peptides (Xiang et al. 2021).

## Initiation of seminal root and postembryonic root meristems

By Frank Hochholdinger and Yaping Zhou

The complex root systems of cereals are composed of different root types formed from meristems at various stages of development (Hochholdinger et al. 2004, 2018). In addition to the primary root whose meristem was discussed in the previous chapter, several cereal species such as maize and barley form a second meristem type in the embryo, which gives rise to seminal roots. A common feature of all cereal root systems is the postembryonic formation of shoot-borne roots that emerge from meristems of below- and aboveground shoot-nodes (Singh et al. 2023). Finally, all cereal root types form postembryonic lateral roots. Lateral roots are all roots that develop from deep inside other roots (Hochholdinger et al. 2018).

In maize, seminal root meristems are formed between 22 and 40 days after pollination in the scutellar node of the embryo (Hochholdinger 2009). To date, 2 genes have been identified that control the formation of seminal root meristems, which both act through auxin. The maize *rtcs* (*rootless concerning crown and seminal roots*) gene (Hetz et al. 1996) controls the initiation of seminal and crown root meristems and encodes a member of the plant-specific family of LATERAL ORGAN BOUNDARY domain (LBD) TFs involved in auxin signal transduction (Taramino et al. 2007 ). *Rootless with undetectable meristem 1* (*rum1*) controls the formation of seminal root meristems in the embryo and of lateral root meristems in primary roots (Woll et al. 2005). The *rum1 gene* encodes an Aux/IAA protein, which is a major component of auxin signal transduction that regulates target genes via interaction with ARF25 and ARF34 (von Behrens et al. 2011).

A recent study surveying >9,000 global maize accessions and their wild relatives uncovered that seminal root number first increased during maize domestication from its progenitor teosinte, followed by a subsequent decrease in response to limited water availability in locally adapted varieties (Yu et al. 2024). Functional characterization of the TF HB77 provided evidence that reshaping root system architecture by reducing the number of seminal roots and promoting lateral root density by modulating the number of meristems of these roots is beneficial for the resilience of maize seedlings to drought (Yu et al. 2024).

Shoot-borne roots in cereals emerge from meristems initiated at consecutive shoot nodes (reviewed in Hochholdinger 2009; Hostetler et al. 2021; Singh et al. 2023), and genetic analyses demonstrated that their formation depends on interactions between auxin and CK related genes. In maize, rice, and wheat, the respective orthologous LBD genes *rtcs* (Taramino et al. 2007), *ADVENTITIOUS ROOTLESS 1/CROWN ROOTLESS 1* (*ARL1/CRL1*) (Inukai et al. 2005; Liu et al. 2005) and *MORE ROOTS* (*TaMOR*) (Li et al. 2022) are master regulators of shoot-borne root initiation. These LBD genes are regulated by AUXIN RESPONSE FACTORS (ARFs; Inukai et al. 2005; Xu et al. 2015; Li et al. 2022). Consistent with the role of auxin signaling in shoot-borne root formation, genes acting upstream of auxin signaling, including auxin biosynthesis, transport, and regulation of transport, have been associated with shoot-borne root formation in rice (Singh et al. 2023).

Upstream of auxin signaling and transport, the micro RNA 156 (miR156), is a conserved regulator of shoot-borne root formation in maize and rice (Chuck et al. 2007; Shao et al. 2019). High levels of miR156 cleave and thus inactivate transcripts of specific TFs of the *SQUAMOSA PROMOTER BINDING PROTEIN-LIKE* (*SPL*) family (Chuck et al. 2007; Shao et al. 2019). In rice, OsSPL3 and OsSPL12 positively regulate the transcription of *OsMADS50* (Shao et al. 2019). In turn, activity of OsMADS50 represses downstream auxin transport and signaling, thus inhibiting crown root formation (Shao et al. 2019). Hence, miR156 cleavage of *OsSPL2* and *OsSPL3* boosts shoot-borne root formation. Similarly, in maize, the *corngrass1* (*cg1*) gene encodes 2 tandem miR156s (*miR156b/c*), which cleave transcripts of multiple *SPL* genes, including *teosinte glume architecture1* (*tga1*) thus enhancing shoot-borne root formation (Chuck et al. 2007).

CK, which acts antagonistically to auxin, is also involved in shoot-borne root formation in cereals. The interplay of auxin and CK is illustrated by the interactions of the auxin-responsive gene *CROWN ROOTLESS 5* (*CRL5*; (Kitomi et al. 2011). *CRL5* encodes a member of the AP2/ERF TF family and is activated by ARF1, similar as *CRL1/ARL1* (Inukai et al. 2005; Liu et al. 2005) and thus acts downstream of AUX/IAA and ARF-mediated auxin signaling (Kitomi et al. 2011). However, *CRL5* and *CRL1*/*ARL1* act in different pathways of shoot-borne root initiation (Kitomi et al. 2011). CRL5 activates the CK responsive genes *OsRR1* and *OsRR2*, which facilitate shoot-borne root initiation by repressing CK signaling. Similarly, OsERF3, another member of the AP2/ERF TF family, activates *OsRR2* expression and thus inhibits CK signaling and promotes shoot-borne root initiation (Zhao et al. 2015b). During crown root emergence and elongation, OsERF3 proteins interact with the TF WOX11 and repress the activity of *OsRR2*. As a consequence, CK signaling is activated and shoot-borne root emergence and elongation is promoted. Hence, while *OsRR2* activity promotes shoot-borne root initiation, repression of *OsRR2* is required for shoot-borne root emergence and elongation. This underscores stage specific differences of CK signaling during shoot-borne root formation (Zhao et al. 2015b). Finally, the rice *CYTOKININ OXIDASE DEHYDROGENASE 4* (*OsCKX4*), which is auxin and CK inducible and a direct target of ARF25, RR2 and RR3, positively regulates shoot-borne root initiation by irreversible degradation of CK (Gao et al. 2014). Recently, it has been shown that *OsCKX4* expression and thus CK degradation is also activated by physical interactions with the TFs WOX11 and CRL1 (Geng et al. 2023). As a consequence, reduced CK levels also reduce WOX11 levels in a negative feedback loop, which decreases the *OsCKX4* transcripts, thus allowing CK to maintain a dynamic balance during shoot-borne root emergence and elongation (Geng et al. 2023).

Lateral root meristems are initiated from specific cell types deep inside the cortical tissue. In maize and rice, pericycle and endodermis cells contribute to lateral meristem formation in a multi-step process of periclinal and anticlinal divisions. In maize, endodermal cells give rise to the epidermis and columella of the newly formed lateral roots, whereas all other cell types of developing lateral roots are formed by pericycle cells at the phloem poles (reviewed in Yu et al. 2016). To date, most maize and rice genes involved in the initiation of lateral root meristems are related to auxin (reviewed in: Yu et al. 2016). The maize gene *rum1* (von Behrens et al. 2011) and the rice genes *IAA11* (Zhu et al. 2012) and *IAA23* (Jun et al. 2011) encode Aux/IAA proteins, which interact with ARF proteins that repress the activity of downstream target genes at low auxin concentrations. At high cellular auxin levels, Aux/IAA proteins are quickly degraded, thus activating transcription of downstream genes related to lateral meristem formation. Several of the *Aux/IAA* genes that control lateral root meristem initiation also affect other aspects of root formation such as seminal root initiation in maize (*rum1*; Woll et al. 2005) or crown root initiation and root cap formation (IAA23; Jun et al. 2011). Moreover, rice CYP2 (CYCLOPHILIN 2) regulates the degradation of Aux/IAA proteins and thus controls asymmetric anticlinal divisions of pericycle cells during lateral root meristem initiation (Kang et al. 2013; Zheng et al. 2013). Furthermore, directional polar auxin transport ensures adequate auxin levels in pericycle and endodermis cells for lateral root meristem initiation. In rice, the auxin influx carrier AUX1 (AUXIN TRANSPORTER PROTEIN 1) is required for lateral root meristem initiation (Zhao et al. 2015a). In addition, the paralogous rice genes *NAL2* (*NARROW LEAF 2*) and *NAL3* that encode WOX3A transcriptional activators, act synergistically in lateral root meristem formation by regulating expression of the auxin transporter genes *PIN1* and *PIN2* (Cho et al. 2013). Finally, the recently cloned *lrt1* (*lateral rootless 1*) gene of maize regulates lateral root meristem initiation via a homolog of the DCAF (DDB1-CUL4-ASSOCIATED FACTOR) protein subunit of the CUL4-based E3 ubiquitin ligase complex (Baer et al. 2023). DCAF proteins are adaptors that bind substrate proteins and promote their ubiquitylation, thus typically marking them for subsequent degradation in the 26S proteasome.

## Initiation and activation of intercalary meristems

By Yongyu Huang

**Figure 4.**
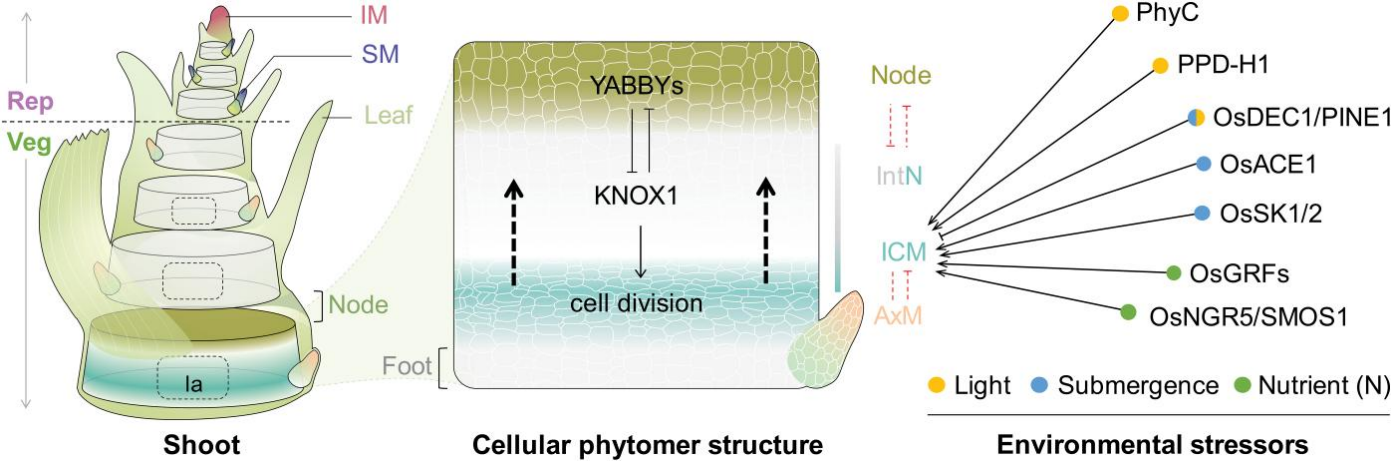
Control of phytomer initiation and ICM activation. The main shoot of vascular plants (barley as a showcase) consists of several repeated phytomers, which originate from the SAM. A gradual shift in the outgrowth of 2 lateral organs—the leaf and the axillary meristem (AxM)—marks the transition from vegetative (Veg) to reproductive (Rep) growth. In cereals, the AxM becomes a spikelet meristem (SM) in reproductive growth. The antagonistic YABBY–KNOX1 regulatory module determines nodes and internodes in a phytomer, which also includes a foot (non-elongating domain at the base) and the ICM. Cell division of the ICMs then displaces cells upward to form an internode (IntN). TFs are highlighted that can respond to various environmental stressors to activate the ICM and induce internode elongation. la, lacuna; —> activation; ——| repression; ------| negative correlation.

In cereal crops, stems are segmented by several morphological hallmarks known as nodes, from which lateral organs—including leaves and axillary buds (or tillers)—emerge. Elongation of the internode between 2 adjacent nodes is controlled by the intercalary meristem (ICM), a group of cells at the base of the internode that undergoes intense cell divisions. The node, lateral organs, and the internode form a structural unit called the phytomer (Fig. 4) (Forster et al. 2007). The presence of ICMs and nodal structures in the cereal stems sets a clear distinction from the model species Arabidopsis in stem development, where cells in the internodes originate exclusively from the rib zone of the shoot tip and extend basipetally (top-down) following transition to reproductive growth. Cell divisions in cereal ICMs, which can span several phytomers away from the shoot tip, extend internodes acropetally (bottom-up) and sequentially, leading to the formation of a large air space known as the lacuna in the central pith region of the internode (McKim 2019). In this way, stem development in cereal crops represents a morphogenetic process that can be broadly divided into 2 phases: (1) phytomer initiation, which originate from the apical meristems to specify the node and internode patterning; and (2) phytomer elongation, which is regulated by the activation of the ICM and is the main determinant of plant height.

How are ICMs established, and what is their function in the context of phytomer units? Previous studies on architectural traits in cereal crops have commonly observed 2 inverse trait relationships: (1) one between phytomer number and length; and (2) another one between internode elongation and axillary bud outgrowth in a phytomer (Fig. 4). This suggests that phytomer initiation and elongation are developmentally integrated, though the detailed mechanisms are not well understood. Architecturally, different organ anlagen in a phytomer are arranged in a specific spatial order: a leaf at the top, followed by a node (lower-half), an internode (includes also the intercalary meristem), root initials, and an axillary bud at the bottom (Fig. 4). Ontogenetically, clonal tracing experiments in maize and rice have revealed that these anlagen share a common cell lineage within the shoot, with the internode cell fate being established last, following the emergence of cells committed to an axillary bud (McDaniel and Poethig 1988; Tsuda et al. 2023). These elegant studies support the concept of a phytomer unit in cereals and imply a presumable constant developmental potential in 1 phytomer unit that is further shared by different organs or tissues (e.g. ICMs vs axillary buds or nodes) in that unit. Indeed, a study in rice identified that antagonistic activity of *KNOTTED_LIKE HOMEOBOX* (*KNOX*) and *YABBY-TFs* forms a module that controls node–internode patterning (Tsuda et al. 2024). High expression of the *YABBY* genes *TONGARI-BOUSHI1* (*TOB1*) and *TOB2* in leaf primordia and nodes specifies their identities; TOB1/2 expression in the internode is repressed by the KNOX gene *OSH15*, allowing for internode formation. Consequently, stems in *osh15* mutant plants have enlarged nodes but shortened internodes, which can be partially recovered by the loss of *TOB1*/*2* (Tsuda et al. 2024). Given the conserved role of *KNOX* genes in maintaining meristem integrity across vascular plants, the KNOX-YABBY module may represent a molecular gateway for designing ideal plant architecture through the modulation of node–internode patterning and ICM establishment in cereal crops.

ICM activation and internode elongation in crops strongly depends on phytohormone metabolism and/or signaling, such as gibberellins (GA), brassinosteroids (BR), and auxin (McKim 2020; Awale and McSteen 2023; Nagai and Ashikari 2023). After reproductive transition, these phytohormones act by stimulating cell division in the ICMs, presumably due to inflorescence-derived signals that reprogram the transcriptome (Gómez-Ariza et al. 2019). Still, little is known about how the elongations of different internodes along the stem axis are developmentally coordinated. For instance, the barley stem typically consists of 4 to 9 internodes of varying lengths, with proximal internodes usually being shorter and distal (upper) internodes extending more significantly until anthesis. Notably, the elongation pattern does not follow a linear trend; instead, it exhibits an “inverse S-shape” regardless of the internode number (Huang et al. 2024). Such a nonlinear elongation pattern is conserved in grasses such as maize (Morrison et al. 1994) and Setaria (Dale et al., 2023). This suggests the presence of yet unknown endogenous and/or external driving forces that influence the basipetal diffusion of inflorescence-derived signals directing their heterogeneous action towards ICM activation (Huang and Schnurbusch 2024). Here, we attempt to summarize external cues that drive the heterogeneous activation of ICMs and internode elongation, considering both above- and below-ground factors (Fig. 4).

Light signals, especially changes in red (R) to far-red (FR) light ratios under dense canopies, can significantly influence intercalary meristem activity and internode length. For instance, wheat plants tend to elongate their proximal internodes more while the distal ones (e.g. the peduncle) become shorter under canopy shade (Golan et al. 2023). Similarly, in maize and rice, more proximal internodes show greater sensitivity towards an elongation response to high planting densities (Song et al. 2016; Zhong et al. 2020). Changes in R/FR ratios are perceived by the phytochrome photoreceptors (Phys), and functional characterization of the wheat photoreceptors *PhyB* or *PhyC* provided evidence that light signals can differentially modulate the elongation of internodes along the stem axis (Kippes et al. 2020). A recent study in barley further demonstrated that internode elongation along the stem axis are regulated by distinct genetic mechanisms, with the photoperiod sensor *Photoperiod 1* (*PPD-H1*) playing a more significant role in controlling the elongation of proximal internodes under long-day conditions (Huang et al. 2024). These examples suggest that the vertical light gradient within a crop plant community may shift the genetic underpinnings of elongation across different internodes.

Another related example is the submergence stress that induces the rapid elongation of specific internodes in certain deepwater rice varieties (Ayano et al. 2014). Under submergence, several additional environmental gradients, such as light, oxygen availability, and osmotic pressure, presumably create a local optimum for intercalary meristem activation. Indeed, the zinc finger TF gene *DECELERATOR OF INTERNODE ELONGATION 1*/*PREMATURE INTERNODE ELONGATION 1* (*DEC1/PINE1*), which is a negative regulator of internode elongation, is repressed under both submergence and shortened light exposures (shift from long day to short day), allowing for rapid internode elongation under environmental fluctuations (Gómez-Ariza et al. 2019; Nagai et al. 2020). DEC1/PINE1 acts antagonistically with ACCELERATOR OF INTERNODE ELONGATION 1 (ACE1) to induce internode-specific GA responses and ICM activation (Nagai et al. 2020). Another early physiological response under submergence is the accumulation of the gaseous phytohormone ethylene due to limited gas diffusion underwater. This induces the expression of 2 AP2/ERF TFs SNORKELs (SK1 and SK2), which then trigger remarkable internode elongation via GA (Hattori et al. 2009).

Soil nutrients, especially nitrogen (N), are key below-ground factors that are crucial for plant growth. In the Triticeae crops wheat or barley, it has been shown that 75% to 90% of the plant's total N uptake occurs during the stem elongation phase (Delogu et al. 1998), suggesting a tight link between N uptake and internode elongation. In fact, selections of semi-dwarf mutations in wheat and rice during the “green revolution” are associated with reduced sensitivity to N-mediated plant height increases (Li et al. 2018). Consequently, rice *semidwarf1* (*sd1*) or wheat *Reduced Height* (*Rht*) alleles inhibit N uptake, which can be overcome by high expression of the TF *GROWTH-REGULATING FACTOR 4* (*OsGRF4*) (Li et al. 2018). Another example is the AP2 TF *SMALL ORGAN SIZE1* (*SMOS1*), also known as *NITROGEN-MEDIATED TILLER GROWTH RESPONSE5* (*NGR5*), which is highly expressed in both node and ICMs to promote internode elongation (Aya et al. 2014; Qiao et al. 2017). It was later reported to reduce sensitivity to N-mediated plant growth when mutated (Wu et al. 2020). Hence, it is likely that dwarfism caused by the inactivation of the ICM is generally associated with reduced sensitivity to N-mediated internode elongation.

In summary, while it is clear that an ontogenetic shift in ICM activation plays a fundamental role in environmental adaptation, we are just beginning to understand how developmental heterogeneity intersects with environmental gradients. This heterogeneity ensures that elongation and growth support are tailored to specific internode regions, allowing for efficient resource allocation and structural adaptation that optimally supports grain production in the inflorescence.

## Establishment and function of inflorescence and other higher-order meristems

By Venkatasubbu Thirulogachandar, Maria von Korff, Shuangshuang Zhao, and Thorsten Schnurbusch

**Figure 5.**
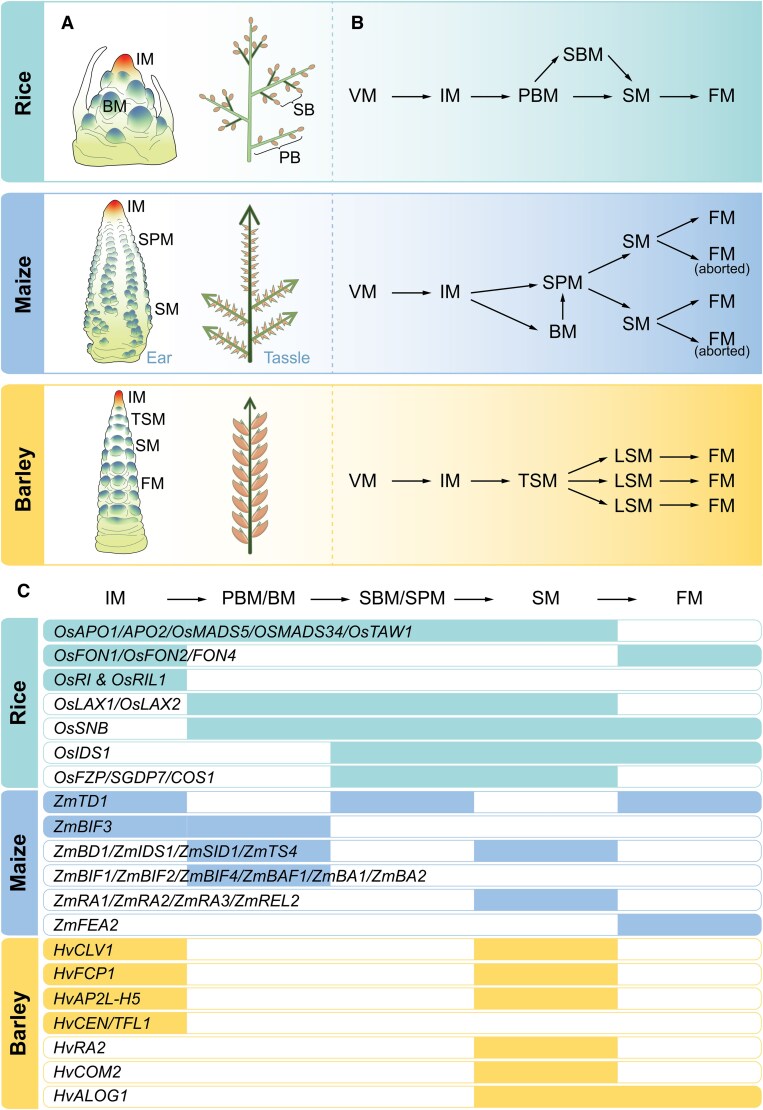
Differences in IMs of cereals. **A)** Schematic illustrations of immature and mature inflorescences of rice, maize, and barley, respectively. The immature inflorescence of rice includes the IM and BM), while the mature inflorescence displays the primary branches (PB), secondary branches (SB), and spikelets. In maize, the immature ear inflorescence shows the IM, spikelet meristem (SM), and SPM, whereas the mature tassel inflorescence depicts the long branches (indicated by side arrows) with the paired spikelets. The immature inflorescence of barley demonstrates the progression of reproductive meristems, encompassing the IM, TSM, 3 SMs, and 3 FMs from the apex to the base. In contrast, the mature inflorescence exhibits 2 rows of spikelets. **B)** Developmental trajectories from vegetative meristem (VM) to FM in rice, maize, and barley are illustrated. **C)** A common differentiation pathway from cereal IM to FM is shown, followed by the genes expressed in various meristems along the pathway for rice (cyan blue), maize (blue), and barley (orange).

The inflorescences of cereal crops exhibit a wide diversity in their structure and form, primarily due to branching patterns, which play a major role in shaping inflorescence architecture. To achieve this complexity, grass inflorescences evolved shifts for specific meristem identities that are associated with diverse shapes and architectures (McSteen et al. 2000; Whipple 2017). The inflorescences of cereals can basically be categorized as either branched-like in rice (panicle) and maize male inflorescence (tassel) or unbranched as maize female inflorescence (ear) and the inflorescence of barley (spike) (Fig. 5A) (Bommert and Whipple 2018; Kellogg 2022; Koppolu et al. 2022). Importantly, the trajectory of inflorescence development in these cereals differs substantially. For example, in rice and maize tassels, the IM generates cells that are destined to form branch meristems (BM) on their flanks. In rice, the primary BM (PBM) later gives rise to secondary BMs (SBMs). The BM of the maize tassel, known as Long BM (LBM), forms a special meristem called “spikelet pair meristem” (SPM), also referred to as short BM (Kellogg 2007; Li et al. 2022). Although the maize ear and barley spike are unbranched, the maize ear IM produces SPM. In contrast, barley's IM generates cells that differentiate into a meristem termed the triple spikelet meristem (TSM) (Fig. 5B). Subsequently, the SBM of rice and SPM of maize generate spikelet meristems (SM). Eventually, SMs of all cereal crops develop 1 or multiple floret meristems (FM). The establishment and function of the above meristem types determine the architecture of the inflorescence and potential grain number. Here, we explore the role of genes and gene networks in shaping the establishment and function of IM and higher-order meristems in 3 major cereal crops: rice, maize, and barley (Fig. 5C).

### Inflorescence meristems (IMs)

The vegetative SAM transitions to the reproductive SAM or IM when FLOWERING LOCUS T-like genes activate this process at the shoot apex (Yan et al. 2006; Komiya et al. 2009; Meng et al. 2011). In Arabidopsis, FLOWERING LOCUS T (FT) is expressed under long-day conditions in the leaves and moves as a protein via the phloem to the SAM, where it interacts with the basic leucine zipper (bZIP) TF FLOWERING LOCUS D (FD) and 14-3-3 proteins to activate the expression of meristem identity genes and induce the transition from a vegetative SAM to a reproductive IM (Abe et al. 2005; Corbesier et al. 2007). The family of *FT*-like genes has expanded by gene duplications occurring independently in nearly all modern angiosperm lineages, including cereal monocots such as barley (12 FT paralogs), rice (13 FT paralogs), and maize (15 FT paralogs) (Chardon and Damerval 2005; Faure et al. 2007; Danilevskaya et al. 2008; Halliwell et al. 2016). While the potential of *FT*-like genes to promote reproductive development is largely conserved in angiosperms, functional diversification has also been demonstrated in cereals. In rice, 2 FT-like paralogs, HEADING DATE3a (Hd3a) and Rice FT1 (RFT1), act as photoperiod-specific florigens. Hd3a is induced under SD conditions to promote flowering, in contrast to its closest homolog, RFT1, which activates reproductive development under LD conditions (Kojima et al. 2002; Tamaki et al. 2007). Hd3a and RFT1 at the shoot apical meristem activate FLOWERING LOCUS T-LIKE 1 (FT-L1), encoding a florigen-like protein that potentiates the effects of Hd3a and RFT1 during the conversion of the vegetative SAM into an IM and organizes panicle branching by imposing increasing determinacy to distal meristems (Giaume et al. 2023). Similarly, in barley, FT3 is expressed under SDs and LDs and induces the transition to an IM and spikelet initiation, while FT1 and FT2 are only expressed under LDs and promote floral development (Digel et al. 2015; Mulki et al. 2018; Shaw et al. 2019). In maize, ZEA CENTRORADIALIS 8 (ZCN8) was identified as the functional homolog of Arabidopsis FT, which promotes floral transition in day-neutral temperate maize and photoperiod-sensitive tropical maize (Danilevskaya et al. 2008; Lazakis et al. 2011; Meng et al. 2011). FT-like genes have thus diversified in cereal monocots to control different stages of reproductive development in response to photoperiod.

The first notable difference in IM function is the emergence of its higher-order meristems—either BM or SM on the main axis (rachis). Rice and maize with branched inflorescences develop BM arranged in a spiral phyllotaxy, contrasting with the distichous arrangement of leaves observed before the transition (Itoh et al. 2005; Chuck et al. 2010; Bartlett and Thompson 2014). In unbranched barley inflorescences, the IM forms TSMs in an alternate-distichous pattern similar to that seen during the vegetative stage (Bartlett and Thompson 2014).

Mutations in the barley *HvCLAVATA1* (*Hv*CLV1) increase the size of the IM at the initiation of spikelet formation, which correlates with the formation of an additional row of SMs in later stages and a shift from a distichous to spiral phyllotaxis at the tip of the inflorescence (Vardanega et al. 2025). Similarly, mutations in the maize ortholog of *CLV1*, known as *THICK TASSEL DWARF1* (*TD1*), result in the enlargement of the meristem and fasciated maize inflorescences, with extra rows of kernels (Bommert et al. 2005). In rice, mutations in the CLV1 homolog *FLORAL ORGAN NUMBER1* (*FON1*) cause enlargement of the floral meristem, an increase in the number of all floral organs and the formation of ectopic floral organs (Suzaki et al. 2004). While the role of CLV1 orthologs in limiting IM size is thus conserved, it only controls phyllotaxis in barley but not rice and maize (Suzaki et al. 2004; Bommert et al. 2005; Moon et al. 2006). However, the CLV3 ortholog of rice FON2/FON4 promotes the spiral phyllotaxy of BMs on the IM (Chu et al. 2006; Suzaki et al. 2006) and neither candidate of *ZmCLE7* and *FON2-like CLE PROTEIN1* (*ZmFCP1*) from maize, nor barley *HvFCP1*, affect IM phyllotaxy (Liu et al. 2021; Vardanega et al. 2025). In rice, mutants for ABERRANT PANICLE ORGANIZATION1 (APO1) or APO2 alter the spiral phyllotaxy of IM to distichous (Ikeda et al. 2005; Ikeda-Kawakatsu et al. 2012). Moreover, mutations in the paralogs VERTICILLATE RACHIS (RI) and RI-LIKE1 (RIL1), which encode BELL1-type homeodomain TFs, result in the formation of BMs in a partially whorled phyllotaxy (Ikeda et al. 2019).

Additional genes have been identified that affect IM size or activity. The panicles of loss-of-function mutants of *OsMADS34* (belonging to the *SEPALLATA*-family of MADS TFs), and double mutants of *OsMADS34* and its paralog *OsMADS5* display increased primary, secondary, and higher-order branches. Consequently, these mutants develop more spikelets (both matured and arrested) compared with the wild type, indicating that these genes contribute to the function of IM and BM and their shift to SM identity (Zhu et al. 2022). An opposite effect was observed in the rice *TAWAWA1* (*TAW1*) mutant plants: *TAW1* is an ALOG family gene, and knockout mutants or RNA interference plants form a small inflorescence, characterized by fewer primary and secondary branches (Yoshida et al. 2013). Similarly, the rice *APO1* and *APO2* mutants generate smaller inflorescences with fewer BM and SM, due to conversion of IMs to terminal SMs culminating in a short panicle (Ikeda et al. 2005; Ikeda-Kawakatsu et al. 2012). A comparable phenotype was observed in the *HvAPETALA2*-like TF (*Hvap2l-H5*) mutant of barley, which forms a short spike with a terminal spikelet (Zhong et al. 2021). To date, no loss of IM indeterminacy mutants have been reported in maize.

In Arabidopsis, an accelerated IM transition and the formation of a terminal flower are observed in mutants of *TERMINAL FLOWER1* (*tfl1*) and plants overexpressing FT (Bradley et al. 1997; Kardailsky et al. 1999; Kobayashi et al. 1999). TFL1 functions as a competitor of FT in binding to FD and 14-3-3 proteins in the shoot apex, thereby preventing the induction of flowering (Ahn et al. 2006). In barley, mutants for *CENTRORADIALIS* (*HvCEN*)/*TERMINAL FLOWER1* (*TFL1*), also display increased IM determinacy, resulting in a shorter spike with fewer spikelets/florets, but do not form a terminal spikelet (Bi et al. 2019 ). RICE CENTRORADIALIS (RCN1 and RCN2) and RCN1-CORN CENTRORADIALIS/TFL1-like protein *ZCN2*, *ZCN4*, *ZCN5* also increase IM indeterminacy, delay flowering, and enlarge tassels and panicles, suggesting that these TFL1 homologs promote indeterminacy by enhancing the functions of both IM and BM (Nakagawa et al. 2002; Danilevskaya et al. 2010).

### Branch meristems (BMs)

Inflorescence development or the shift to higher-order meristem identity (Fig. 5B) can be viewed as a continuous branching process that ends when the FM develops floral organ primordia on its axis (Kellogg 2022). In general, all higher-order meristems of the inflorescence are axillary meristems (AxM) that develop at the axil of a suppressed bract (Galli et al. 2015; Wang et al. 2021b). Unlike the IM, the first-order BM of rice (PBM) and maize tassel (LBM) exhibit either a distichous, a biased distichous, or a spiral arrangement of lateral meristems (SBM in rice and SPM in maize), which indicates that a dynamic molecular process controls the phyllotaxy of the first-order BM in cereals. Notably, all BMs and their SMs develop their lateral meristems in a distichous arrangement (Itoh et al. 2005; Chuck et al. 2010; Bartlett and Thompson 2014; Chen and Gallavotti 2021; Du et al. 2022). Finally, FM branches out the floral organ primordia in a whorl arrangement (Bartlett and Thompson 2014; Strable and Vollbrecht 2019).

The genes mentioned earlier that influence the IM activity in rice—*OsMADS5*, *OsMADS34*, *TAW1*, *APO1*, and *APO2*—also extend their control over the BM and SM, suggesting that interconnected molecular processes govern the higher-order meristems in rice. In maize, however, several genes and pathways that impact the establishment and function from BMs to other higher-order meristems have been identified. Mutations in the *BARREN STALK* (*BA*) and *BARREN INFLORESCENCE* (*BIF*) genes in maize have revealed key contributions to the development of axillary or branch meristems in inflorescence. In these mutants, the BMs are either completely suppressed or develop randomly on the inflorescence; notably, in the *BIF3* mutant, the size of the IM is increased alongside the absence of BMs. *BIF1* and *BIF4* encode AUX/IAA proteins, while *BIF2* encodes a Serine/Threonine Protein Kinase known as PINOID, which regulates auxin transport (McSteen and Hake 2001; McSteen et al. 2007; Galli et al. 2015). However, *BIF3* harbors a tandem duplicated copy of the *ZmWUSCHEL1-B* gene, resulting in gene overexpression mediated by CK signaling TFs (Chen et al. 2021). The *BA1* and *BA2* mutants are orthologs of rice's *LAX PANICLE1* (*LAX1*) and *LAX2* genes, which encode a basic helix-loop-helix TF that acts downstream of auxin. The *LAX1* mutant in rice influences only the reproductive axillary meristems, while *LAX2* extends even to the vegetative AxM (Komatsu et al. 2003; Gallavotti et al. 2004; Tabuchi et al. 2011; Yao et al. 2019). Collectively, these mutants highlight the crucial roles of auxin and CK in establishing BMs in both maize and rice. Another maize mutant, *BARREN STALK FASTIGIATE1* (*BAF1*), revealed an essential gene for the proper formation of the ear meristem and the angle of branching in long tassel branches. This gene codes for an AT-hook DNA binding motif TF and can form dimers with BA1 (Gallavotti et al. 2011).

The *RAMOSA* (*RA*) mutants in maize provide insight into the determinacy of the short branch meristems, SPM, and the angle of long branches in tassels. In the *RA1* (Vollbrecht et al. 2005), *RA2* (Bortiri et al. 2006), and *RA3* (Satoh-Nagasawa et al. 2006) mutants, the SPM of the ear and tassel develops into a long branch meristem. *RA1 ENHANCER LOCUS2* (*REL2*) significantly enhances the phenotypes observed in *RA1* and *RA2* mutants. It is reported that RA2 and RA3 act upstream of RA1 (Gallavotti et al. 2010). In barley, mutations in RA2 (HvRA2, VRS4) show increased lateral spikelet fertility and the development of TSMs into secondary inflorescence-like structures, demonstrating that HvRA2 promotes TSM determinacy and represses lateral spikelet fertility in barley (Koppolu et al. 2013).

### Spikelet meristems (SMs)

In grasses, a “small spike” is a specialized short branch called spikelet that consists of 1 or more florets; spikelets are the basic reproductive units. The SM is subsequently able to initiate 1 or more floret meristems (FMs), with each producing sexual floral organs such as carpels, stamens, and lodicules, along with a grain-bearing floret. Meristem determinacy is acknowledged as essential for the development of diverse inflorescence architectures. For instance, a wheat spikelet is indeterminate, enabling it to produce multiple lateral florets within each spikelet. In contrast, a barley spikelet is determinate, resulting in the formation of a single-floret spikelet.

Although SM identity and determinacy are conceptually distinct, genes frequently influence both traits simultaneously. For example, cloning of the maize *BRANCHED SILKLESS1* (*BD1*) gene provided the first example for a SM identity regulator. *bd1* mutants show altered identity of the SM, causing indeterminate branch- or inflorescence-like structures to form in the place of spikelets (Chuck et al. 2002). The orthologous genes in rice (*FRIZZY PANICLE*, *FZP*) and barley (*COMPOSITUM2*, *COM2*) have a very similar role in promoting SM identity (Komatsu et al. 2003; Poursarebani et al. 2015). It is interesting to note that the ear phenotypes of the 5 *bd1* alleles varied in severity, with the weak allele forming fewer branches and a few fertile florets, while the more severe alleles converted nearly all initial spikelets into indeterminate branch-like structures. Similarly, natural variations in the upstream sequences of *SMALL GRAIN AND DENSE PANICLE* (*SGDP7*) and *CONTROL OF SECONDARY BRANCH1* (*COS1*), 2 different *FZP* alleles, increase branch number and grain yield (Bai et al. 2017; Huang et al. 2018), suggesting that an optimal balance between BM and SM can be sought through the regulation of BD1/FZP/COM2 to boost grain yield. In contrast to promoting SM identity, rice APOs inhibit the acquisition of SM identity. ABERRANT PANICLE ORGANIZATION1 (APO1) (ortholog of Arabidopsis UNUSUAL FLORAL ORGANS [UFO]) physically interacts with RICE FLORICAULA LEAFY (RFL)/APO2 (ortholog of Arabidopsis LEAFY) (Ikeda et al. 2005; Ikeda-Kawakatsu et al. 2012). The cooperation of APO1/2 towards inhibiting SM fate acquisition, and both *apo1* and *apo2* mutations caused the precocious conversion of the SM identity, exhibiting reduced branch numbers. Short Panicle 3 (SP3) modulates SM identity in a manner similar to APO1/2 by promoting APO2 expression (Huang et al. 2019). In contrast, LARGE2 interacts and degrades APO1/2, and promotes SM fate acquisition (Huang et al. 2021). The rice ALOG TF TAW1 negatively regulates the acquisition of SM identity through the control of *SVP*-family MADS-Box genes (Yoshida et al. 2013), while barley ALOG1 specifies SM identity and determinacy via non–cell-autonomous signals from the boundary-domain of the SM (Jiang et al. 2024).

SM determinacy, namely the number of florets produced, is determined by the SM itself following the acquisition of SM identity. Genetic evidence indicates that the miR172-AP2 module regulates SM determinacy. In maize, INDETERMINATE SPIKELET1 (IDS1) acts redundantly with a close paralog, SISTER OF IDS1 (SID1), to regulate SM determinacy. Both *ids1* and *sid1* mutants produce more than the regular 2 florets (Chuck et al. 1998, 2008). Notably, *TASSELSEED4* (*TS4*) encodes a miR172, which targets the IDS1 and SID1, highlighting the crucial role of miR172 in regulating SM determinacy (Chuck et al. 2007). *Os*INDETERMINATE SPIKELET1 (*Os*IDS1) and SUPERNUMERARY BRACT (SNB), the orthologs of IDS1 and SID1, have similar roles in controlling SM determinacy, and *snb*, *snb osids1*, and *Os*miR172-overexpressing lines all produce extra florets (Lee and An 2012).The IDS1 ortholog present in barley *Hv*AP2L-H5 plays a role in SM determinacy and represses the formation of extra florets and terminal SM (Zhong et al. 2021). It is intriguing to note that the miR172-AP2 module controls not only SM determinacy but also IM determinacy, suggesting that fine-tuning these genes to extend IM activity could be a potential strategy for increasing spikelet number. In fact, the miR172-AP2 module also affects FM establishment and floret organ development. Finally, barley mutants for *Hv*FCP1 or *Hv*CLV1 can form an extended axis bearing 2 FMs, revealing yet another role for CLE peptides and their receptors in an SM determinacy pathway (Vardanega et al. 2025).

### Floret meristems (FMs)

The FM develops florets and produces grains after fertilization. Mutations of the maize and TD1/FEA2 and rice FON1/FON4/2 result in an increase in the number of floral organs (Taguchi-Shiobara et al. 2001; Suzaki et al. 2004, 2006; Bommert et al. 2005). *OsMADS1*, one of the *SEPALLATA-LIKE* MADS-box genes, has been unraveled to play specific roles in rice FM identity and FM determinacy. Mutants of *OsMADS1* perturb cell differentiation in the lemma and palea that result in abnormalities such as glume-like structures, reduced stamen number, the formation of malformed carpels, or an extra floret within the spikelet (Prasad et al. 2005). In barley, HvMADS1 is expressed in SMs and subsequently lemmas, paleae, marking the SM initiation and lemma/palea identity (Zhong et al. 2021). Furthermore, under high ambient temperatures, HvMADS1 is responsible for maintaining an unbranched spike architecture, while the loss-of-function mutant forms a branched inflorescence-like structure (Li et al. 2021). HvMADS1 directly regulates the CK-degrading enzyme HvCKX3 to integrate temperature response and CK homeostasis, which is required to repress meristem cell cycle/division.

In summary, while several genes are known to regulate the formation of a spikelet by affecting the meristem identity transition, how to leverage this knowledge to benefit grain yield production remains a challenge. Many meristem identity and/or determinacy genes are indispensable for proper plant architectural development, and their complete knockout often results in undesirable developmental abnormalities, compromising overall plant growth and productivity. Future genome editing of the cis-regulatory regions in these genes may allow more subtle changes in quantitative traits that are most desirable for production (Rodríguez-Leal et al. 2017).

## Stem cells shape grass leaves

By Lea Berg and Michael Raissig

**Figure 6.**
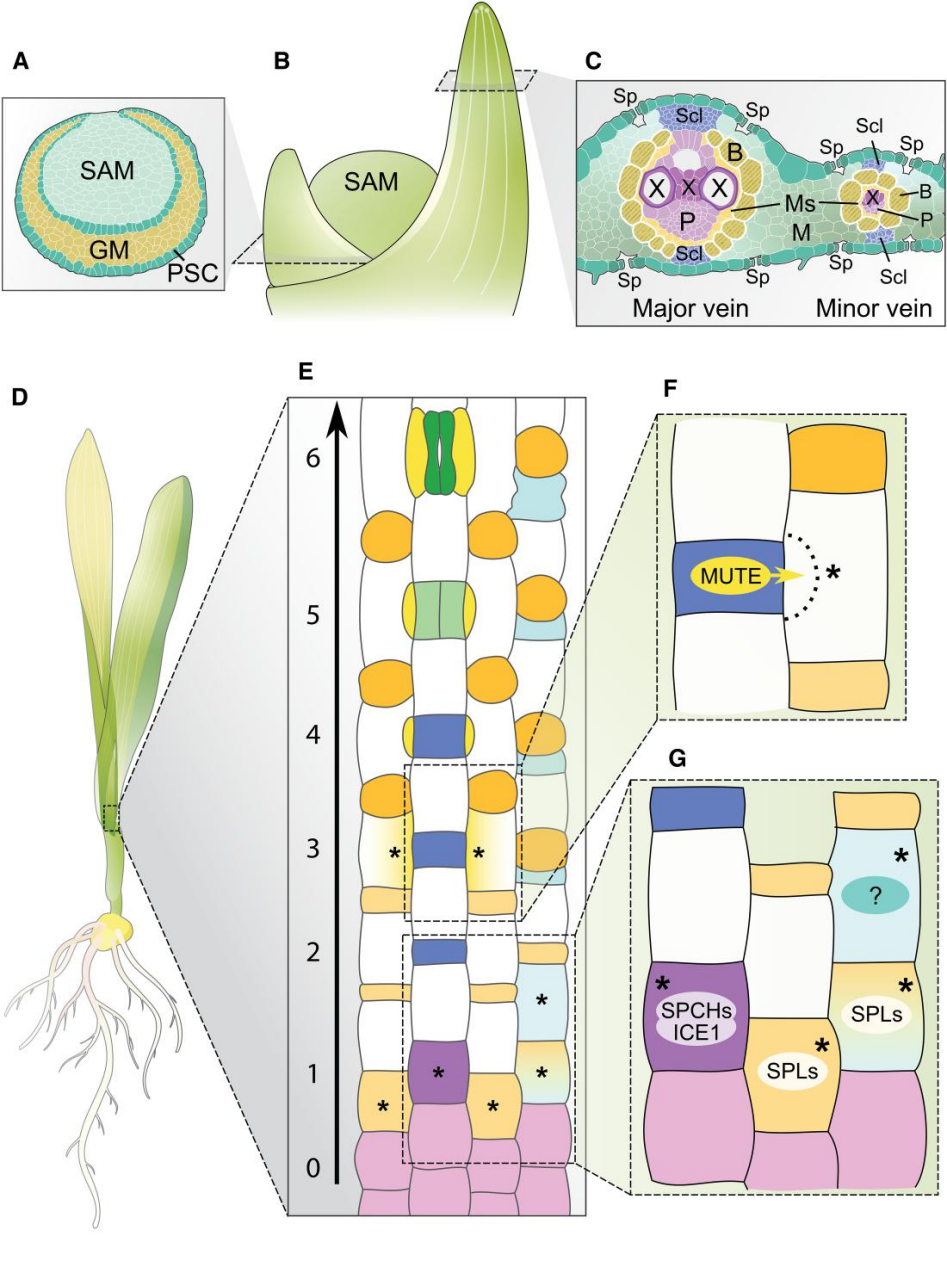
Stem cell systems during leaf development. **A)** Schematic of a cross-section through a young leaf primordium with the inner ground meristem (GM) and outer protodermal stem cells (PSC). The primordium envelops the vegetative SAM. **B)** Schematic of the SAM with a young (left) and an older (right) leaf primordium. **C)** Schematic of a cross-section through the mature leaf zone with a major and minor vein. Vein tissues include xylem (X), phloem (P), mestome sheath (M) and bundle sheath cells (B). Sclerenchymatic cells (Scl) are indicated above and below the veins and the outermost cell layers are epidermal cells with stomatal pores (Sp) adjacent to the veins. **D)** Schematic of a young grass seedling. **E)** Schematic of the leaf epidermal developmental zone. Seven developmental stages are shown with protodermal cells (PSCs, stage 0), and the 6 stages of stomatal development from lineage establishment to mature stomatal complexes (stage 1–6); asterisks indicate cells that retain division capacity beyond the PSC stage (0). **F)** Magnification of the stomatal subsidiary mother cell (SMC, asterisk). The TF BdMUTE is expressed in the guard mother cell (GMC, blue) and moves into lateral neighbor cells to establish SMC identity and induce an asymmetric division to form subsidiary cells (SCs, indicated by dashed line). **G)** Magnification of the developmental stages 0 to 2. Cells with an asterisk divide asymmetrically in transverse orientation to form stomatal precursors, hair cell precursors or silica cell precursors, respectively. The different TFs specifying cell file identities are indicated.

Grass leaves have 2 main units, the proximal leaf sheath and the distal leaf blade (Fig. 6) (Conklin et al. 2019; Richardson et al. 2021). The blade provides the majority of photosynthetic activity and plant-environment gas exchange. The sheath envelops the outgrowing younger leaves at the base of the leaf and provides protection and stability. It allows grass shoots to grow upward without hypocotylar extension, thus protecting the vegetative SAM (Kaplan 1973; Johnston et al. 2015; Conklin et al. 2019; Richardson et al. 2021).

The development of grass leaves is initiated with the formation of leaf primordia at the SAM (Fig. 6, A and B) (Kaplan 1973; Johnston et al. 2015; Conklin et al. 2019; Satterlee and Scanlon 2019; Richardson et al. 2021). The leaf primordium fully encircles the SAM and different zones along the mediolateral axis contribute to different parts of the leaf (Fig. 6A). Within each primordium exist 2 SCNs, the protodermal stem cells (PSCs) in the L1 layer that form leaf epidermal cells and the inner ground meristem (GM) in the L2, which will differentiate into leaf vasculature and mesophyll (Fig. 6, A and C) (Dengler et al. 1985; Trivett and Evert 1998; Sakaguchi and Fukuda 2008).

Early within the leaf primordia, major veins are patterned and differentiated from the ground meristem, whereas minor vein formation occurs later during primordium and leaf elongation (Nelson and Dengler 1997; Baird et al. 2021; Robil and McSteen 2023). All leaf vein cell types derive from transversal and longitudinal divisions of a procambial initial cell (Perico et al. 2022). The positioning and initiation of future leaf veins is linked to auxin maxima and auxin flow away from tips is thought to connect new veins to existing plant vasculature (Lee et al. 2009; O’Connor et al. 2014; Johnston et al. 2015; Robil and McSteen 2023). PIN1a is among the earliest markers expressed in procambial cells and future veins, but it has been suggested that vein positioning might be prepatterned before the accumulation of PIN1a and, consequently, auxin flux (Robil and McSteen 2023; Perico et al. 2024). Upon procambial establishment and PIN1a activation, a vein developmental program is sequentially activated to form all tissues of mature veins and guarantee water and solute transport (Fig. 6C) (Perico et al. 2024). Whether the interspersed mesophyll development is the default state and primarily induced by the absence of the vein program remains unclear.

PSCs in the outermost L1 layer form all specialized leaf epidermal cell types like stomata, hair cells, pavement cells, silica cells, and bulliform cells (Fig. 6, A and E) (Kellogg 2016). The grass leaf epidermis is organized in linear cell files with pluripotent PSCs at the leaf base (stage 0, Fig. 6, D and E) and differentiated, mature cells towards the leaf tip (Stebbins and Shah 1960; Nunes et al. 2020). Early in development, PSC pluripotency is terminated, and cell files commit to distinct cell lineages (Stage 1, Fig. 6E). In *B. distachyon*, cell files can either generate pavement and hair cells, or pavement and stomatal guard cells. While many of the early patterning programs remain unknown, some core regulators in establishing the stomatal or the hair cell lineage were identified (Fig. 6, E and G). The specific position of stomatal complexes next to leaf veins likely involves a SHORTROOT (SHR) and SCARECROW (SCR) TF module, which also patterns the root and shoot vasculature (Slewinski et al. 2012; Schuler et al. 2018; Hughes et al. 2019; Hughes and Langdale 2022). Expression of maize SHR in the rice vasculature induced ectopic stomatal rows (Schuler et al. 2018) and OsSCR is among the earliest markers of stomatal rows (Kamiya et al. 2003a). Stomatal lineage establishment requires the SPEECHLESS bHLH TFs (SPCH1 and SPCH2) and INDUCER OF CBF EXPRESSION 1 (ICE1/SCRM) (Fig. 6G) (Raissig et al. 2016; Wu et al. 2019). In *B. distachyon*, *bdspch1 bdspch2* double mutants and *bdice1* single mutants lack stomatal complexes, which were seemingly replaced by hair cells (Raissig et al. 2016). In contrast, hair cell fate is established by SPL TFs in maize and rice (Fig. 6G) (Lan et al. 2019; Kong et al. 2021; Liao et al. 2023). In rice, SPLs seem to interact with OsWOX3B to coordinate hair cell fate (Angeles-Shim et al. 2012; Kong et al. 2021 ; Liao et al. 2023). Strikingly, the *zmspl10 zmspl14 zmspl26* triple maize mutant lacks hair cells and forms ectopic stomata instead. Together, this suggests that both hair cell and stomatal fate need to be actively established and repress each other for proper epidermal patterning (Raissig et al. 2016; Kong et al. 2021).

Upon cell lineage commitment, a single transverse asymmetric division generates a small apical cell and a large basal cell (stage 2, Fig. 6E). The small apical cell will become the specialized cell (i.e. stomatal guard cells or hair cell), while the large cell usually forms a pavement cell (Fig. 6, D and E). This causes the within-file patterning characteristic of the grass family, where specialized cell types, such as guard cells or hair cells, are always spaced by at least 1 pavement cell (Liu et al. 2022). This within-file patterning is controlled by a grass-specific BRX-solo polarity gene and the conserved patterning MAP triple kinase YODA1 in both barley and *B. distachyon* (Abrash et al. 2018; Liu et al. 2022).

Even though PSCs are terminated, and the mediolateral patterns are established in stage 1 of epidermal development, the large daughter cells of stage 2 asymmetric divisions retain a significant level of pluripotency to flexibly pattern additional epidermal cell types. For example, in stages 3 and 4, such large daughter cells are reprogrammed to subsidiary mother cells (SMCs) by the mobile MUTE TF, which moves from guard mother cells to large lateral neighbor cells (Raissig et al. 2017; Wang et al. 2019 ; Spiegelhalder et al. 2024). SMCs resemble secondary, adult stem cells as they can perform longitudinal asymmetric divisions to form stomatal subsidiary cells more than once if they flank multiple guard mother cells (Fig. 6, E and F) (Raissig et al. 2017; Wang et al. 2019). Similarly, in some large daughter cells (mostly above veins), additional transverse asymmetric divisions can form other specialized small cells like silica or cork cells that are usually associated with hair cells or each other (Fig. 6, E and G) (Liu et al. 2022). How the sustained (or re-established) division capacity and the switch in cell identity post-division is regulated remains enigmatic. In addition, the genetic programs establishing and differentiating the leaf-rolling bulliform cells remain a mystery. Finally, mediolateral and within-file patterns are highly diverse within the grass family and comparative analyses of early grass leaf development to dissect the rewiring of epidermal patterning modules are still lacking.

## Initiation and function of male and female stem cell-like systems

By Martina Balboni, Arp Schnittger, Jia-Chi Ku, and Karina van der Linde

In contrast to animals, plants do not set up a stem cell system during early embryogenesis to generate a germline, which then generates gametes during the entire or parts of the lifetime of the organism (Bhatt et al. 2001). Instead, plants initiate a germline de novo from somatic cells in female and male floral organs produced by the IM (Goldberg et al. 1993). This transition typically starts in mature plants and depends on both environmental as well as developmental factors (see above). Inside the female and male floral organs, the megasporangium (the ovule) and the microsporangium (also called anther or pollen sac) are formed, respectively. The development of the female and male organs can take place in the same flower, as seen in Arabidopsis, barley, and *Brachypodium*, or occurs in a spatially and temporally separated manner, for example in maize, giving rise to separated male and female flowers with distinct morphologies (diclinous) on the same plant (monoecious) (Cheng et al. 1983; Dellaporta and Calderon-Urrea 1993).

### Stem cell-like systems in the female germline

**Figure 7.**
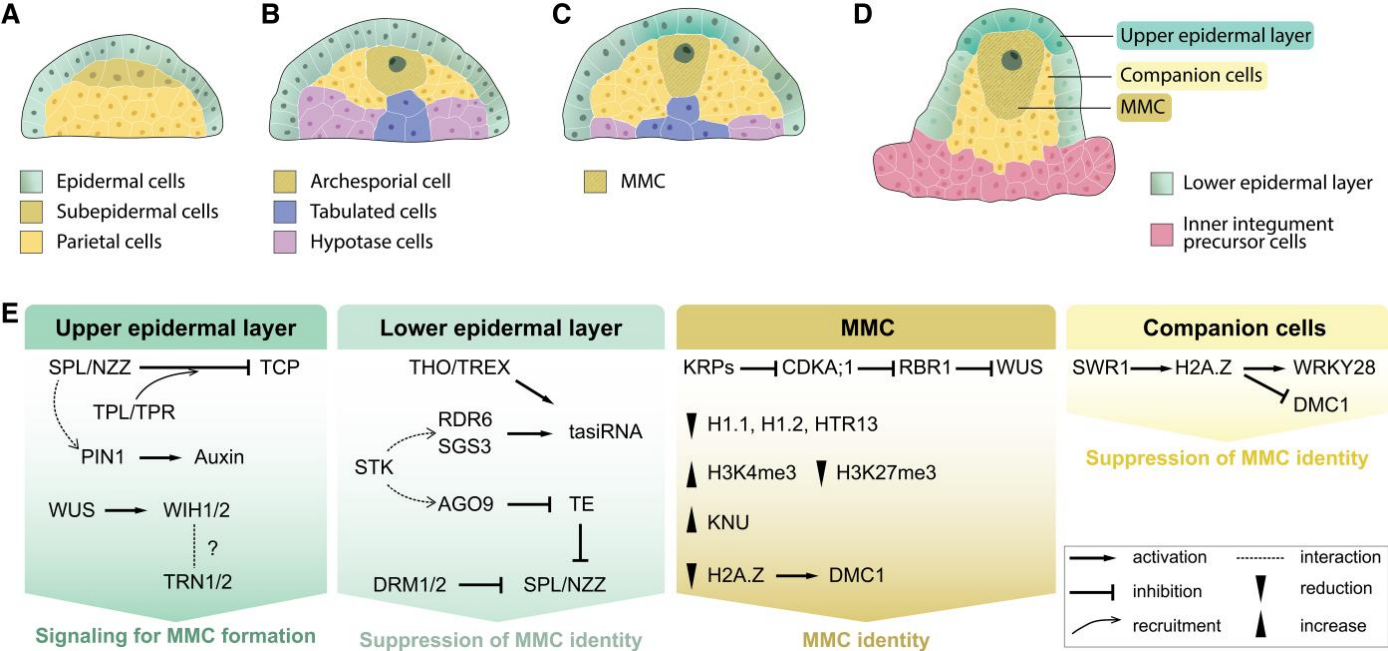
Female germline specification. Sketch of megaspore mother cell (MMC) formation in maize (A to C) and key pathways involved in MMC specification in Arabidopsis (D and E). **A)** The archesporium is formed at the nucellar region of the ovule primordium and likely involves a stem cell-like system. **B)** Only 1 subepidermal cell per ovule primordium acquires the reproductive fate, the archesporial cell (AR). Adjacent to the AR, the ovule primordium contains several cell types which presumably support the developing germline. **C)** The archesporial cell develops into a female meiocyte, i.e. the MMC. Surrounding cell types are indicated. **D)** Ovule primordium of Arabidopsis containing an MMC. Additional cell types can be distinguished. **E)** Key pathways involved in MMC specification in Arabidopsis: the upper epidermal layer promotes the establishment of an environment suitable for MMC formation, while the lower epidermal layer together with the companion cells suppresses MMC identity. Pathways to acquire and maintain MMC identity are also indicated. See text for details of molecular players and pathways.

During megasporangium formation, several cell types need to be specified. Foremost, a group of subepidermal cells, representing the area of the future germline, forms from which then one, the archesporial cell, adopts reproductive fate (Fig. 7, A and B). The archesporial cell (AR) either produces or differentiates into the megaspore mother cell (MMC), i.e. the designated meiocyte (Walbot and Egger 2016; Pinto et al. 2019; Böwer and Schnittger 2021) (Fig. 7C). The generation of the AR represents the beginning of the germline. Besides the AR and the MMC, the ovule primordia hold several other cell types: in maize, these are the parietal cells, the tabulated cells and the hypotase cells, which can be morphologically defined and presumably support the developing germline and gametophyte (Voronova et al. 2003). Female germline development then continues with the MMC undergoing meiosis, a specialized cell division that leads to a reduction in chromosome number, accomplished by 2 consecutive nuclear divisions without an intervening DNA replication phase. In addition, new allelic combinations are generated by the new assortment of chromosome sets and homologous recombination (Mercier et al. 2015; Zickler and Kleckner 2015). Meiosis concludes the life cycle of the sporophyte and marks the beginning of the gametophytic phase, during which the gametes form.

The female meiotic products are the megaspores, of which only one, typically the most basal one, survives and forms the functional megaspore (Yang et al. 2010; Grossniklaus 2011). The functional megaspore undergoes 3 nuclear divisions (polygonium type) in many species such as maize, followed by cellularization resulting in an eight-celled embryo sac, the mature female gametophyte. The embryo sac contains 2 female gametes, egg cell and central cell, respectively (Yang et al. 2010; Erbasol Serbes et al. 2019; Pinto et al. 2019). The fertilized egg cell develops into an embryo while the fertilized central cell gives rise to the endosperm, a nourishing tissue that supports embryo growth during the subsequent stages of seed development (Yang et al. 2010; Pinto et al. 2019).

### Reprogramming cell fate to initiate the female germline

A key question for (female) reproductive development in all plant species including cereals is how somatic, mitotically dividing cells adopt germline fate (Schmidt et al. 2015). Work from Arabidopsis has suggested that the adoption of the germline program involves the formation and action of stem-cell like cells including the stem cell factor WUS and its sister proteins (Fig. 7, D and E; reviewed in (Lora et al. 2019; Pinto et al. 2019; Böwer and Schnittger 2021; Cai et al. 2022; Kaur et al. 2024). WUS is also required for the formation of MMCs in Arabidopsis (Gross-Hardt et al. 2002; Zhao et al. 2017c). Consistently, *WUS* was found to be expressed in the apical part of young ovule primordia. However, later during ovule development, WUS is absent in the MMC and only resides in the epidermal layer above (Tucker et al. 2012; Zhao et al. 2017c). The repression of *WUS* in the MMC is executed by the Arabidopsis homolog of the human tumor suppressor protein RETINOBLASTOMA RELATED 1 (RBR1), which can bind to the promoter of *WUS* (Zhao et al. 2017c). In somatic cells, RBR1 is a crucial repressor of cell proliferation. In mitotically dividing cells, RBR1 is phosphorylated by the action of cyclin-dependent kinases (CDKs) in conjunction with their cyclin partners (Weimer et al. 2012). For entry into meiosis, CDKs were found to be inactivated by a class of redundantly acting CDK inhibitors, named KIP-RELATED PROTEINs (KRPs) due to their partial sequence similarity with the mammalian CDK inhibitor p27^Kip1^ (Zhao et al. 2017c). Loss of KRP function, similar to the reduction of RBR1 activity, causes the accumulation of WUS in the MMC and is connected with the execution of a mitotic division program in the designated MMC. This causes the formation of multiple MMCs, of which remarkably at least some can develop into functional supernumerary embryo sacs within 1 ovule. Importantly, the formation of multiple MMCs is partially suppressed through the reduction of WUS activity highlighting the importance of WUS regulation for female germline development (Zhao et al. 2017c) (Fig. 7E).

Currently, it is not clear whether the action of WUS and the presumptive formation of stem-cell like cells is instrumental for a trans-differentiation process (e.g. stem cell fate may erase all differentiation cues of somatic cells, e.g. chromatin marks, allowing the subepidermal cells to be competent for signals inducing gametic fate) or whether WUS in conjunction with other factors directly promotes germline fate. On the one hand, the finding that the expression of *WUS* needs to be repressed in a cell designated to undergo meiosis may argue for the former scenario. On the other hand, 2 WUS targets have been identified in ovule primordia, WINDHOSE 1 (WIH1) and WIH2, which are required for proper MMC differentiation suggesting a direct role of WUS in germline specification (Lieber et al. 2011). How KRP expression is directed to germline precursor cells to ultimately allow the repression of *WUS* is not clear. A potential regulator is the predicted TF SPOROCYTELESS/NOZZLE (SPL/NZZ), which appears to function upstream of WUS. In the corresponding *spl/nzz* mutant, MMCs are not formed (Yang et al. 1999; Balasubramanian and Schneitz 2000; Lieber et al. 2011; Wei et al. 2015; Mendes et al. 2020). Whether the function of WUS and its regulators is conserved in cereals is not clear, but WUS, WIH1, and WIH2 homologs are found in maize. Maize expresses at least 2 *WUS* and 3 RB-related genes, which still need to be functionally characterized during female germline development (Nardmann and Werr 2006; Sabelli and Larkins 2009; Chen et al. 2023).

A common principle between cereals and Arabidopsis might be that germline fate is progressively restricted to 1 cell. Apparently, many components are engaged in this restriction in Arabidopsis (Fig. 7E) and likely other plants since mutants in many different pathways cause the formation of supernumerary MMCs or MMC-like cells including, for example, mutants affected in the formation of small RNAs. Conversely, these mutants also indicate that several subepidermal cells have the potential to initiate a germline. These germline competent cells might compete with each other through a lateral inhibition mechanism as suggested by the analysis of mutants in *MULTIPLE ARCHESPORIAL CELLS1* (*MAC1*) in maize. In *mac1*, several subepidermal cells develop into ARs and then MMCs (Sheridan et al. 1996). MAC1 encodes for a small protein and during male germline formation, MAC1 was found to be secreted (Wang et al. 2012). Based on the sequence similarity and the mutant phenotype, MAC1 appears to be the ortholog of rice TDL1A, previously described to be the ligand of a membrane-localized LRR receptor-like protein kinase (LRR-RLK) called MSP1 (MULTIPLE SPOROCYTE1) (Nonomura et al. 2003; Zhao et al. 2008), and the ortholog of Arabidopsis TPD1 (Canales et al. 2002; Zhao et al. 2002; Yang et al. 2005; Jia et al. 2008). Since it is usually the central most subepidermal cell which will acquire germline fate, it is tempting to speculate that there is positional information besides a lateral inhibition mechanism and/or that this positional cue provides a bias to the lateral inhibition mechanism. Possibly, such a positional bias is provided by the phytohormone auxin. Auxin accumulates at the tip of the nucellar epidermis in Arabidopsis, most likely via the accumulation of PIN1 and PIN3 in the epidermis (Benková et al. 2003; Pagnussat et al. 2009; Ceccato et al. 2013). Conversely, CK has been detected in the basal part of the Arabidopsis ovule (Zürcher et al. 2013). Notably, the presence of auxin in the epidermal layer of Arabidopsis overlaps with other regulators such as WUS and WIH1/2, and NZZ/SPL was also found to be required for PIN1 expression. An apical auxin maximum has also been reported in maize (Forestan et al. 2012; Lituiev et al. 2013), suggesting the presence of a conserved microenvironment around the female germline and a role for the epidermal cells in transmitting the signal. Moreover, the distal end of the MMC in maize expands more than the proximal end, possibly hinting at an effect of auxin.

Taken together, the picture of how the entry into female germline is controlled is still very mosaic. Several components and pathways have been identified in Arabidopsis (Fig. 7E), but it is unclear how they act together and to what degree they are conserved in cereals and other plants. There probably exist species-specific differences as the entry into both female and male germline is, for example, affected in *msp1* mutants in rice (Nonomura et al. 2003), but such a female mutant phenotype was not reported in Arabidopsis. Given the importance of female reproductive development, for instance in the context of apomixis, it is urgently necessary to understand the molecular mechanism of entry into the female germline in cereals.

### Stem cell-like systems in the male germline

**Figure 8.**
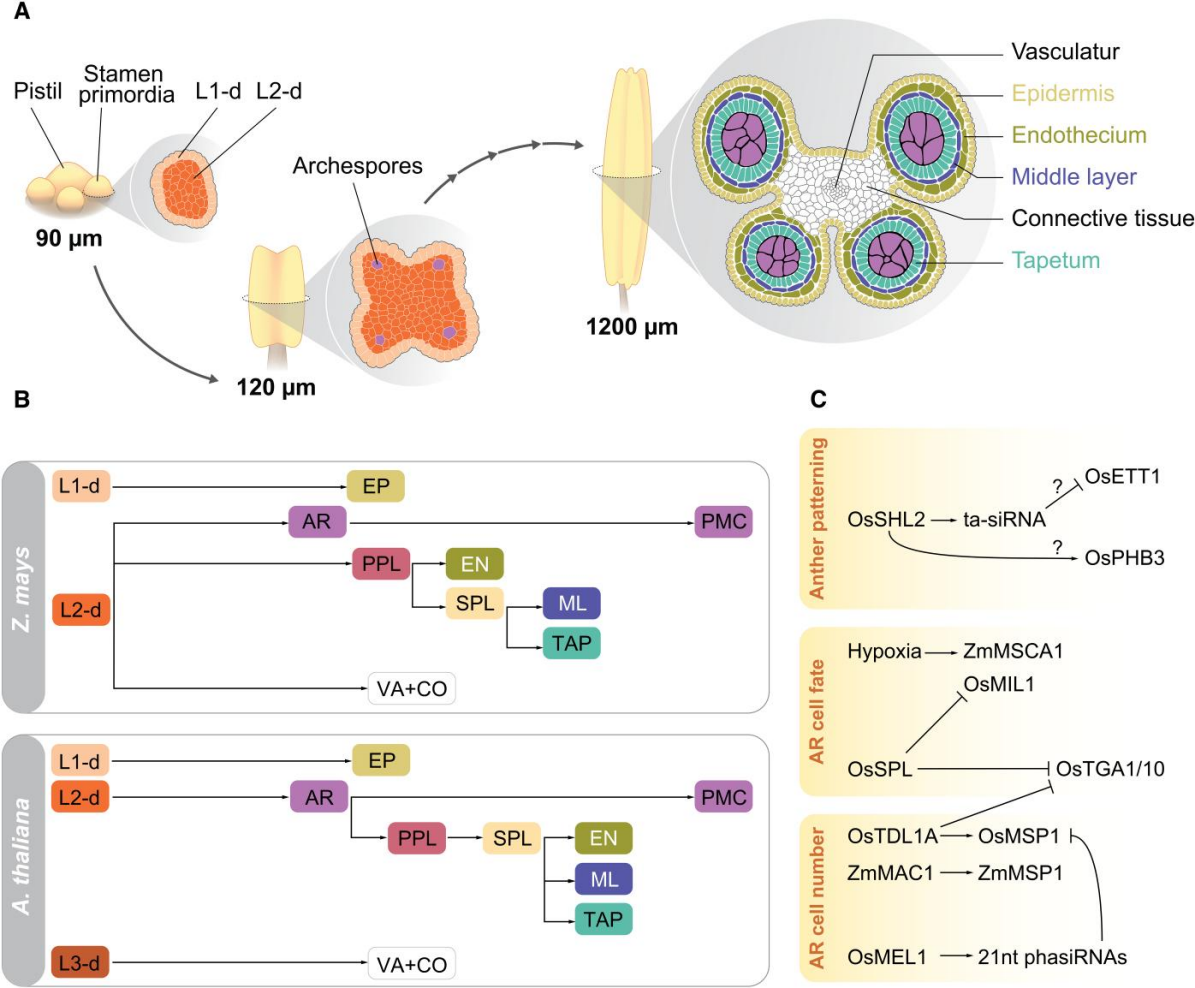
Developmental progression and regulatory networks in male germline specification. **A)** Schematic representation of anther development in maize as an example. Early stages involve the differentiation of L1 and L2-d layers (anther length 90 *µ*m), with the emergence of archespore cells (ARs) at an anther length of 120 *µ*m. Subsequent stages lead to the formation of somatic layers, including the tapetum (TAP), middle layer (ML), endothecium (EN), epidermis (EP), and vasculature (VA), shown in cross-section at 1,200 *µ*m length stage. **B)** Comparative lineage relationships of maize and Arabidopsis anther tissues. L1-d and L2-d layers give rise to different cell types, including the epidermis, primary and secondary parietal cell layers (PPL, SPL), and AR, which later become pollen mother cells (PMC). Note that the formation of somatic anther lobe layers (TAP, ML, EN) has been described by different models in both species. The additional L3-d cells in Arabidopsis contribute to VA and connective (CO) tissues. **C)** Regulators of anther development in maize and rice. See text for details.

Many of the stem cell and stem cell-like systems discussed in this review are highly morphologically and developmentally variable or even unique in cereals compared with model species such as Arabidopsis. In contrast, anthers, which harbor the male germline, have a largely conserved structure in angiosperms. Anther cross sections have a butterfly-like shape, with 2 thecae, each containing 2 lobes, surrounding the connective tissue and vasculature (Fig. 8A). The germline cells, which are positioned in the lobe center, are encircled by 4 somatic cell layers: tapetum, middle layer, endothecium and epidermis. These somatic layers are all essential for proper germline and subsequent pollen development and their dispersal (Kelliher and Walbot 2011; Egger and Walbot 2016).

The FM gives rise to floral organ primordia, including the stamen primordia. In a leaf-like patterning process, axial and abaxial domains are established resulting in the formation of filament and anther primordia (Toriba et al. 2010). The latter contains pluripotent L1-d (meristematic layer 1-derived) stem cells surrounding species-dependent L2-d and/or L3-d stem cells. In maize, all inner anther primordia cells date back to FM L2, whereas in Arabidopsis, both L2-d and L3-d cells contribute to anthers (Fig. 8B). Outgrowth at the primordium corners results in the characteristic four-lobed shape (Raghavan 1988; Toriba et al. 2010). In parallel, L2-d cells acquire germline cell fate and become AR cells in the 4 corners of the anther primordia, and L1-d cells specify into the epidermis. Elegant work in maize using confocal microscopic imaging to trace cell division patterns demonstrated that the AR surrounding L2-d cells undergo a periclinal division to form the endothecium and the secondary parietal layer, which in turn divides into the tapetum and the middle layer, respectively (Fig. 8B) (Kelliher and Walbot 2011). In dicots, it has been postulated that L2-d cells give rise to AR cells, which develop either into the germline or through primary and secondary parietal cells into the somatic tissue layers (Fig. 8B) (Goldberg et al. 1993; Gómez et al. 2015; Åstrand et al. 2021). Notably, in Arabidopsis, the middle layer can be generated from both the inner and outer secondary parietal cells (Xue et al. 2021). Anther lobe layer specification is followed by mitotic proliferation of all anther cells and differentiation of AR cells into pollen mother cells (also called microspore mother cell), i.e. the male meiocyte. After meiosis, the haploid microspores mitotically divide twice to form tricellular pollen.

### Regulators of anther patterning and germline initiation

To date, the initial patterning and shaping of the anther is known to rely on LRR-RLKs, mitogen-activated protein kinases (MPKs), and RNA-dependent RNA polymerase (RDR) involved in trans-acting siRNA (ta-siRNA) pathways (Hord et al. 2006, 2008; Toriba et al. 2010). In rice, *OsETT1* (an ortholog of *AtARF3/ETT*) is first expressed on the abaxial side of the stamen primordia, whereas *OsPHB3* (an ortholog of *AtPHB, PHABULOSA*) is expressed in the adaxial domains (Fig. 8C). During a rapid polarity change, these expression patterns are rearranged into a cross pattern that corresponds to the later theca and lobe structure of an anther. This expression rearrangement is facilitated by the RDR *Os*SHL2, which acts in the ta-siRNA pathway, and subsequently ta-siRNAs mediate the cleavage of *OsETT1* transcripts (Toriba et al. 2010). Prior work in Arabidopsis has revealed similar expression patterns of *AtARF3* and *AtPHB* by in situ mRNA hybridization studies (Sessions et al. 1997; Dinneny et al. 2006). These data indicate that patterning of anther primordia into the 4-lobe anther structure is conserved between monocots and dicots. However, early on transcriptional differences exist within the subepidermal anther cells even though they appear morphologically similar.

Loss of the RLKs *At*BAM1, *At*BAM2, *At*ER/ERL/ERL2, or the protein kinases *At*MPK3/6, which have been proposed to be downstream in the receptor kinase signaling cascade not only results in anther patterning defects but also disrupts male germline development (Hord et al. 2006, 2008; Zhao et al. 2017a). These observations indicate that patterning can influence germline development. However, it remains unclear whether in Arabidopsis the above-mentioned components required for patterning are also directly involved in germline initiation, or whether the resulting early anther form is crucial (e.g. for the gradient formation of germline initiation signals), or a combination of both, and whether these factors also contribute to male germline initiation in cereals.

A limited number of factors have been identified that are directly involved in male germline initiation, thereby terminating the pluripotency of anther primordia cells (Fig. 8C). These factors include a MADS box TF-related protein, naturally occurring hypoxic conditions, a CC-type glutaredoxin, a ligand-receptor module, an ARGONAUTE protein, and small RNAs (Sheridan et al. 1996, 1999; Schiefthaler et al. 1999; Yang et al. 1999; Nonomura et al. 2007; Kelliher and Walbot 2012; Wang et al. 2012; van der Linde et al. 2018; Dukowic-Schulze and van der Linde 2021; Zheng et al. 2021). Disturbance of these factors leads to abnormal development or even abortion of the male germline. The most prominent example is the repressor protein *At*SPL/NZZ (mentioned above already for female germline entry). Its loss results in failure of AR differentiation and defects in the somatic cell layers of the male flower, and absence of meiocytes in the female flower (Schiefthaler et al. 1999; Yang et al. 1999). A functional ortholog of *At*SP/NZZ has been identified in rice (*Os*SPL) (Ren et al. 2018).

For the hypoxia signal, it was proposed that a naturally occurring oxygen gradient within the 4 corners of the anther is sensed by the glutaredoxin *Zm*MSCA1, resulting in the instruction of the innermost lobe cells to adopt AR fate (Kelliher and Walbot 2012). Exposure of maize anthers to elevated hypoxic conditions triggers the development of ARs from L1-d cells (Kelliher and Walbot 2012). Since L1-d cells normally form the epidermis, this result implicates that although L1-d and L2-d cells originate from different meristematic layers, they share the same level of pluripotency and competence (Kelliher and Walbot 2012). Homologs of *Zm*MSCA1 have been identified in rice (MICROSPORELESS1, *Os*MIL1) and Arabidopsis (*At*ROXY1 and *At*ROXY2). Anther development in *Osmil1* mutants is arrested after archesporial, endothecium, and secondary parietal layer formation (Hong et al. 2012). Deletion of *AtROXY1* and *AtROXY2* prevents formation of normal parietal cells and pollen release while meiosis is still induced. Two bZIP TFs (*AtTGA9/10*) are coexpressed with *AtROXY1/2* in anthers and *Attga9/10* mutants phenocopy *Atroxy1/2.* In addition, AtROXY1/2 and AtTGA9/10 directly interact and regulate an overlapping set of anther genes (Murmu et al. 2010). Also in maize, interaction of MSCA1 with a bZIP TF has been reported. Maize male inflorescence development is controlled by redox changes in the bZIP TF FASCIATED EAR4 (FEA4) induced by MSCA1 and 2 paralogous glutaredoxins, leading to activation of FEA4 (Yang et al. 2021). Based on these reports one might speculate that MSCA1 interacts with bZIP TFs and by this transmits the natural occurring hypoxic conditions into the nucleus for AR specification.

Once ARs are specified, they are controlled by the ligand-receptor pair *Zm*MSP1-*Zm*MAC1 in maize (Wang et al. 2012; van der Linde et al. 2018). This regulatory unit is also required for somatic niche formation from L2-d cells. Loss of *Zmmsp1* or *Zmmac1* in maize results in excess ARs and failure of surrounding L2-d cells to differentiate (Wang et al. 2012; Timofejeva et al. 2013; van der Linde et al. 2018). Like *At*ROXY1/2-*Os*MIL1-*Zm*MSCA1, *Zm*MAC1 and *Zm*MSP1 are also conserved in rice (*Os*TDL1A and *Os*MSP1) and the dicot Arabidopsis (*At*TPD1 and *At*EMS1/EXS). Again, mutants display different cytological phenotypes in Arabidopsis, rice, and maize (Sheridan et al. 1999; Nonomura et al. 2003; Yang et al. 2003, 2016; Wang et al. 2012; Huang et al. 2016). These phenotypic differences might lie in small changes in the respective environmental conditions, expression patterns, turnover and cell-to-cell migration properties. For example, Arabidopsis, rice, and maize anthers are of different sizes and in different environments during formation.

As mentioned above, small RNA pathways also are crucial for germline development. Rice *argonaute OsMEL1* (*MEIOSIS ARRESTED AT LEPTOTENE 1*) is expressed in stamen primordia. At later stages of anther development, *OsMEL1* transcripts were detected predominantly in germline cells (Nonomura et al. 2007). *Os*MEL1 functions in the development of pre-meiotic AR cells and binds preferentially to 21-nt phasiRNAs. Based on this, it was proposed that lincRNAs are processed into reproductive 21-nt phasiRNAs via initial cleavage dependent on 22-nt miR2118. *Os*MEL1 then binds to reproductive 21-nt phasiRNAs and assures proper germline development (Komiya et al. 2014). Consistent with the data of Komiya et al. (2014), it was reported recently that *OsMSP1* is targeted by phasiRNAs, per definition becoming then ta-siRNAs (Huang et al. 2020; Zhang et al. 2020); this connects small RNAs and ligand-receptor signaling during pluripotency termination and germline development. Notably, reproductive phasiRNAs are widely presented in flowering plants but appear to be lacking in the Brassicaceae, Fabaceae, and Solanaceae (Pokhrel et al. 2021).

Taken together, the above descriptions point to differences in male germline initiation between cereals and the model plant Arabidopsis, with missing key players, inconsistent phenotypes and different cell origins. Components that regulate anther patterning and germline initiation include small RNA signaling, ligand-receptor modules, and environmental factors. Some of these factors are known regulators of stem cell systems in other parts of the plant. Published data suggest that these components may interact, but to what extent and how they are linked remains largely unclear. Therefore, we hypothesize that monocot-specific but also conserved factors within the anther determine germline initiation and thereby terminate the anther primordia stem cell-like system.

## Conclusions and perspectives

In conclusion, this comprehensive review provides an overview and highlights significant advancements on our current understanding how cereal meristems and their SCNs are established and maintained, how they function, and how the knowledge generated can be applied to optimize grass crop architecture. While many molecular players like core components of the CLE/CLV-WOX as well as auxin and CK signaling pathways previously identified in Arabidopsis appear conserved in cereals, their detailed analysis shows substantial diversification, modification and dynamics. Thus, knowledge about the extent of conservation of underlying pathways are still largely elusive. A number of novel factors including transcriptional regulators and receptor kinases have been identified involved in the establishment of additional and more complex cereal organs such as the root and inflorescence systems, but the underlying GRNs still remain to be identified. Moreover, many molecular players and/or modified pathways are still unknown in cereals which regulate, for example, adaptation of meristem activity to changing environments, nutrient and light signals. The role of small RNA pathways has not been studied intensively, many cell identity genes and regulators of cereal-specific organs are unknown, and regulators of adult and germline stem cells are just emerging.

However, it can be expected that the detailed expression pattern of known and novel players in cereal meristems and their stem cell systems will be elucidated. Single-cell RNA sequencing (scRNA-seq) has now emerged as a tool for uncovering molecular regulations across different plant species. This technique enables the analysis of RNA transcriptomes on a cellular level, providing detailed insights into cell-type-specific gene expression. It also allows investigating transcriptional dynamics in developmental processes and heterogeneous cell populations (reviewed in (Bawa et al. 2022). In Arabidopsis, scRNA-seq studies on roots led, for example, to the identification of 24 cell clusters and their respective marker genes (Zhang et al. 2019). The combination of scRNA-seq data with pseudotime analysis unraveled developmental trajectories from stem cells to differentiated cells, which were characterized by TF expression waves (Denyer et al. 2019). ScRNA-seq and chromatin accessibility studies in rice radicles allowed the reconstruction of developmental trajectories for epidermal cells and ground tissues, revealing expression networks regulating cell fate. In addition, conserved and divergent pathways, for example, in root development could be characterized within eudicotyledons and monocotyledons. Combined with spatiotemporal modeling, this enabled the identification of *Os*GATA6 as a key regulator of root meristem differentiation in rice (Zhang et al. 2021). scRNA-seq of maize root tips, for example, led to the identification of 7 major cell types and 21 transcriptionally distinct clusters as well as immune-regulatory networks (Cao et al. 2023) and cell-by-cell comparative analysis using different C4 cereals demonstrated how fine-scale cellular profiling can extract conserved regulatory modules from a pan transcriptome and provide insight on the evolution of cells that mediate key meristem functions in cereals (Guillotin et al. 2023). Altogether these approaches indicate that scRNA-seq represents a promising technique to significantly accelerate knowledge on cereal meristem function in the future.

Although functional studies in cereals such as barley, wheat and maize have been a bottleneck for many years, transformation methods became more accessible in recent years and methods like CRISPR/Cas9-mediated gene editing are highly efficient in cereals (Ahmar et al. 2023) and can be used, for example, to increase yield potential by manipulation the expression of *CLE* genes (Liu et al. 2021). We expect that our knowledge on cereal meristems will significantly increase in the near future. As indicated above, recent advances in single-cell omics technologies now offer an unprecedented opportunity to delve deeper into the composition of cereal meristems and SCN activities (Thibivilliers and Libault 2021; Denyer and Timmermans 2022). By sequencing single cells from meristems at different developmental stages or environmental conditions, researchers can now identify distinct cell types and numerous cell type-specific genes. When combined with spatial transcriptomics (Xia et al. 2022; Wang et al. 2024) and multiplex hybridization techniques (Joossens et al., 2024; Perico et al. 2024; Wang et al. 2024; Wu et al. 2025), these powerful approaches enable the simultaneous observation of hundreds of gene expression patterns to identify more and novel meristem regulators. These will significantly accelerate our understanding of cereal meristem functions, close knowledge gaps and provide the necessary tools to shape cereal architecture for human needs.

## Data Availability

No new data was generated or analyzed.

## Dive Curated Terms

The following phenotypic, genotypic, and functional terms are of significance to the work described in this paper:

PIN1 Gramene: AT1G73590
PIN1 Araport: AT1G73590

RBR1 Gramene: AT3G12280
RBR1 Araport: AT3G12280

APO2 Gramene: AT5G57930
APO2 Araport: AT5G57930

CLV2 Gramene: AT1G65380
CLV2 Araport: AT1G65380

WOX4 Gramene: AT1G46480
WOX4 Araport: AT1G46480

LSH1 Gramene: AT5G28490
LSH1 Araport: AT5G28490

PIN3 Gramene: AT1G70940
PIN3 Araport: AT1G70940

PIN7 Gramene: AT1G23080
PIN7 Araport: AT1G23080

tpd1 Gramene: AT4G24972
tpd1 Araport: AT4G24972

SPL Gramene: AT4G27330
SPL Araport: AT4G27330

CLV1 Gramene: AT1G75820
CLV1 Araport: AT1G75820

WOX11 Gramene: LOC_Os11g47580
WOX11 Araport: LOC_Os11g47580

ARF1 Gramene: AT1G59750
ARF1 Araport: AT1G59750

AUX1 Gramene: At2G38120
AUX1 Araport: At2G38120

CLE40 Gramene: AT5G12990
CLE40 Araport: AT5G12990

RCN1 Gramene: Os11g0152500
RCN1 Araport: Os11g0152500

TGA1 Gramene: At5g62510
TGA1 Araport: At5g62510

PHYB Gramene: AT2G18790
PHYB Araport: AT2G18790

PHYC Gramene: BRADI1G08400
PHYC Araport: BRADI1G08400

WOX8 Gramene: AT5G45980
WOX8 Araport: AT5G45980

CRL1 Gramene: Os03g05510
CRL1 Araport: Os03g05510

LARGE2 Gramene: LOC_Os12g24080
LARGE2 Araport: LOC_Os12g24080

PINOID Gramene: AT2G34650
PINOID Araport: AT2G34650

WUS1 Gramene: AT2G17950
WUS1 Araport: AT2G17950

SMC Gramene: Soil moisture content
SMC Araport: Soil moisture content
